# Supplementary figures and images for: mTOR activity is essential for retinal pigment epithelium regeneration in zebrafish
Source: PLoS Genet. 2022 Mar 10;18(3):e1009628. doi: 10.1371/journal.pgen.1009628 (PMC8939802; doi:10.1371/journal.pgen.1009628)

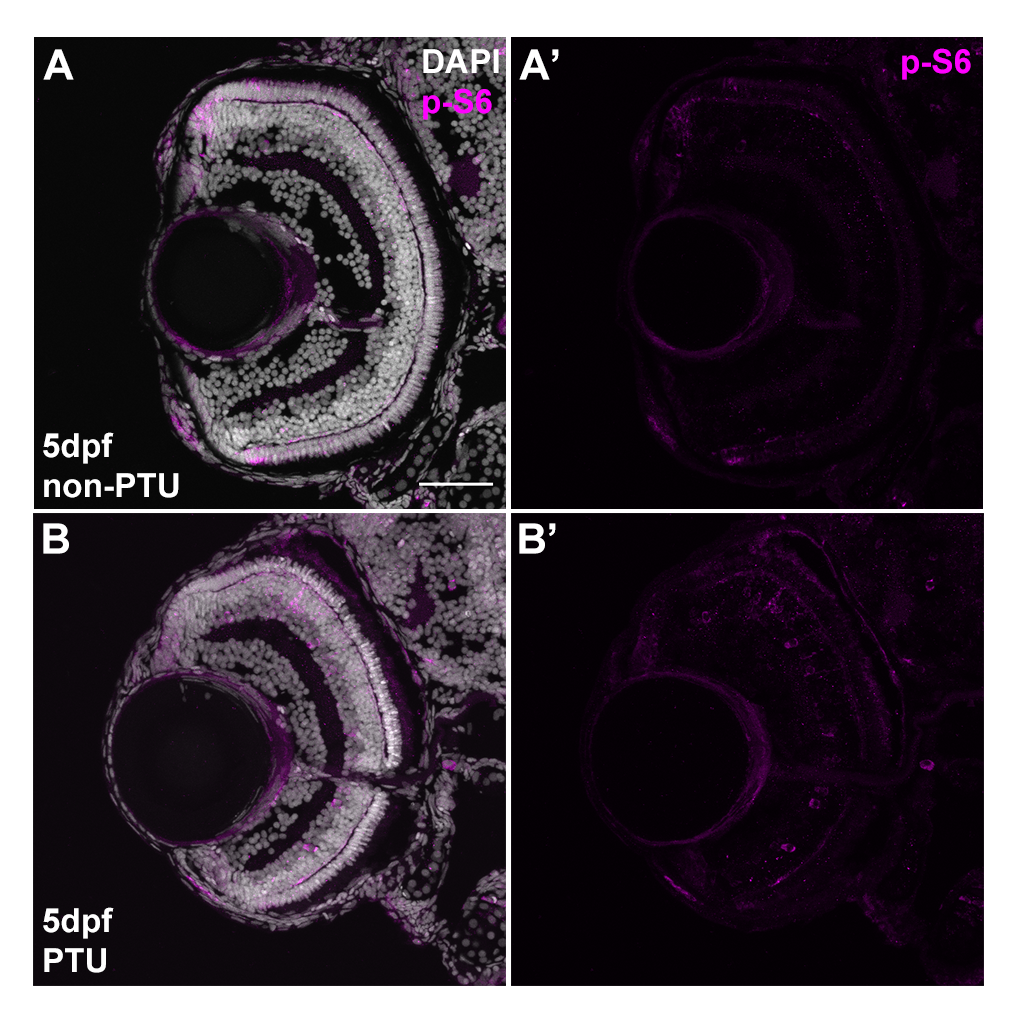

Supplement: S1 Fig — (A-B) Representative fluorescent images of p-S6 staining on cryosections from (A) non-PTU (n = 10) and (B) PTU-treated larvae (n = 10) at 5dpf. (A’-B’) Single channel immunofluorescent images of p-S6. Dorsal is up and distal is left. Scale bar = 50μm. (TIF) [file pgen.1009628.s001.tif]

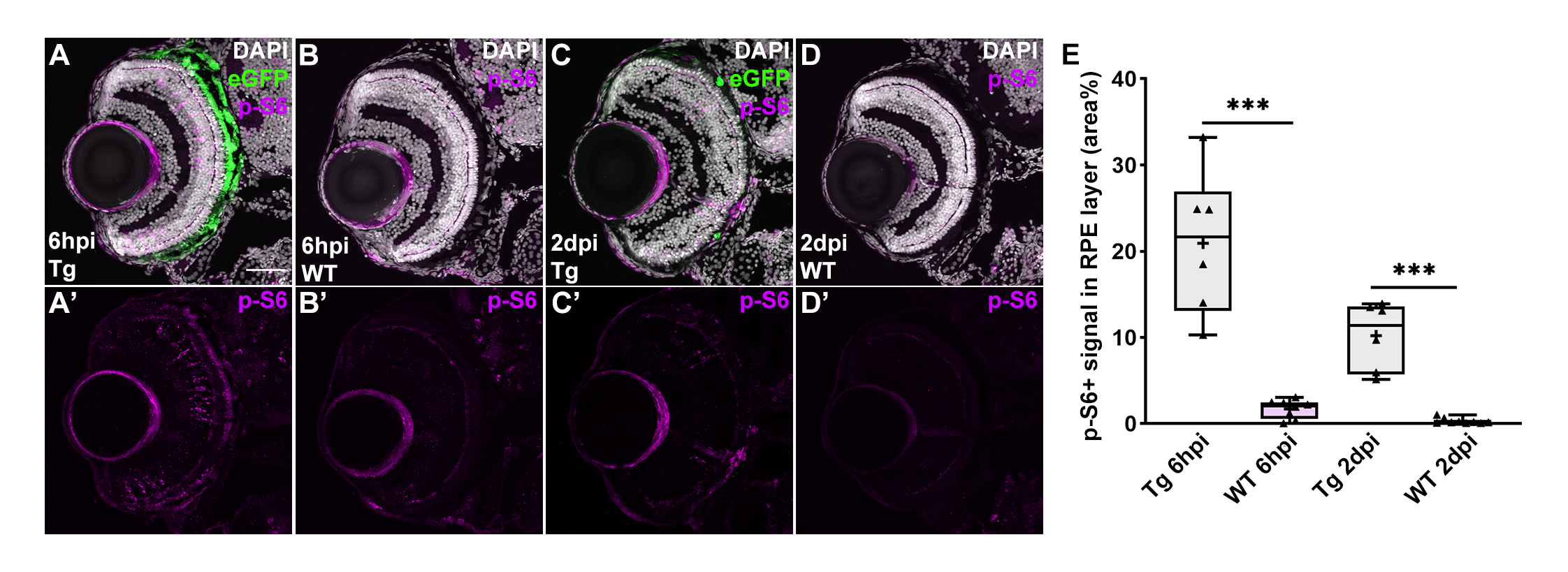

Supplement: S2 Fig — (A-D) Immunofluorescent images of p-S6 staining on transverse cryosections from (A,C) MTZ-treated larvae carrying rpe65a:nfsB-eGFP transgene (MTZ+ Tg) and (B,D) MTZ-treated wild-type larvae (MTZ+ WT). Nuclei (white), p-S6 (magenta), eGFP (green). (A’-D’) Single channel immunofluorescent images of p-S6. (E) p-S6 signals in the RPE layer were significantly elevated post-MTZ treatment (6hpi and 2dpi) in rpe65a:nfsB-eGFP Tg larvae when compared to WT controls. Statistical information can be found in S9 Table. Dorsal is up and distal is left. Scale bar = 50μm. (TIF) [file pgen.1009628.s002.tif]

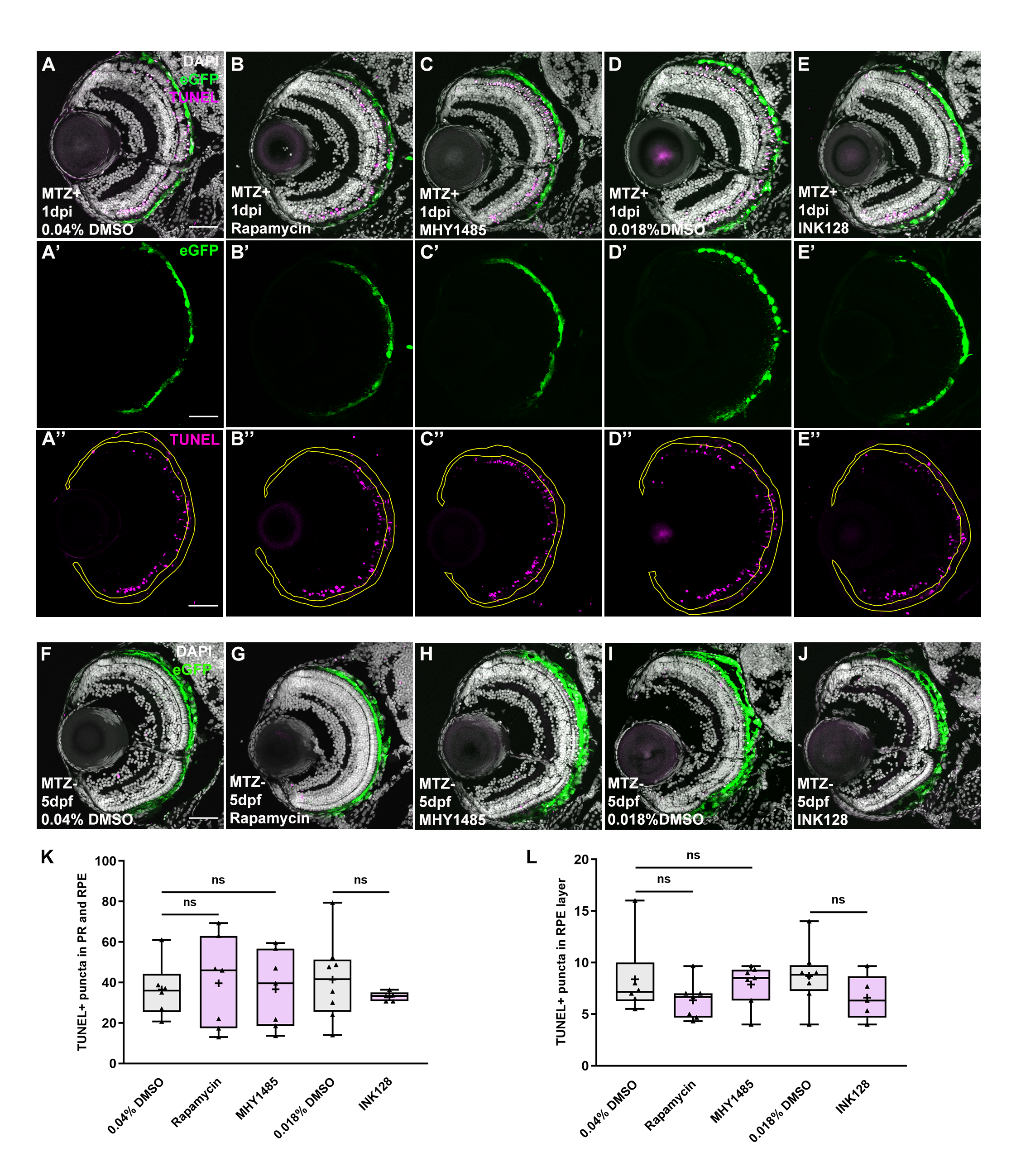

Supplement: S3 Fig — (A-E) Fluorescent images of TUNEL staining on cryosections from MTZ+ 0.04%DMSO-, 2μM rapamycin-, 2μM MHY1485- and 0.018% DMSO and 0.9μM INK-treated larvae at 1dpi. Single channel immunofluorescent images of eGFP (A’-E’) and TUNEL (A”-E”). (F-J) Fluorescent images of TUNEL staining on cryosections from MTZ- 0.04%DMSO-, 2μM rapamycin-, 2μM MHY1485- and 0.018% DMSO and 0.9μM INK-treated larvae at 6dpf. Quantification of TUNEL+ puncta between the outer plexiform layer and RPE layer (K) and solely RPE layer (L) showed no significant differences between DMSO-treated and drug-treated groups. Statistical information can be found in S9 Table. Nuclei (white), eGFP (green), TUNEL (magenta). Dorsal is up and distal is left. Scale bar = 50μm. (TIF) [file pgen.1009628.s003.tif]

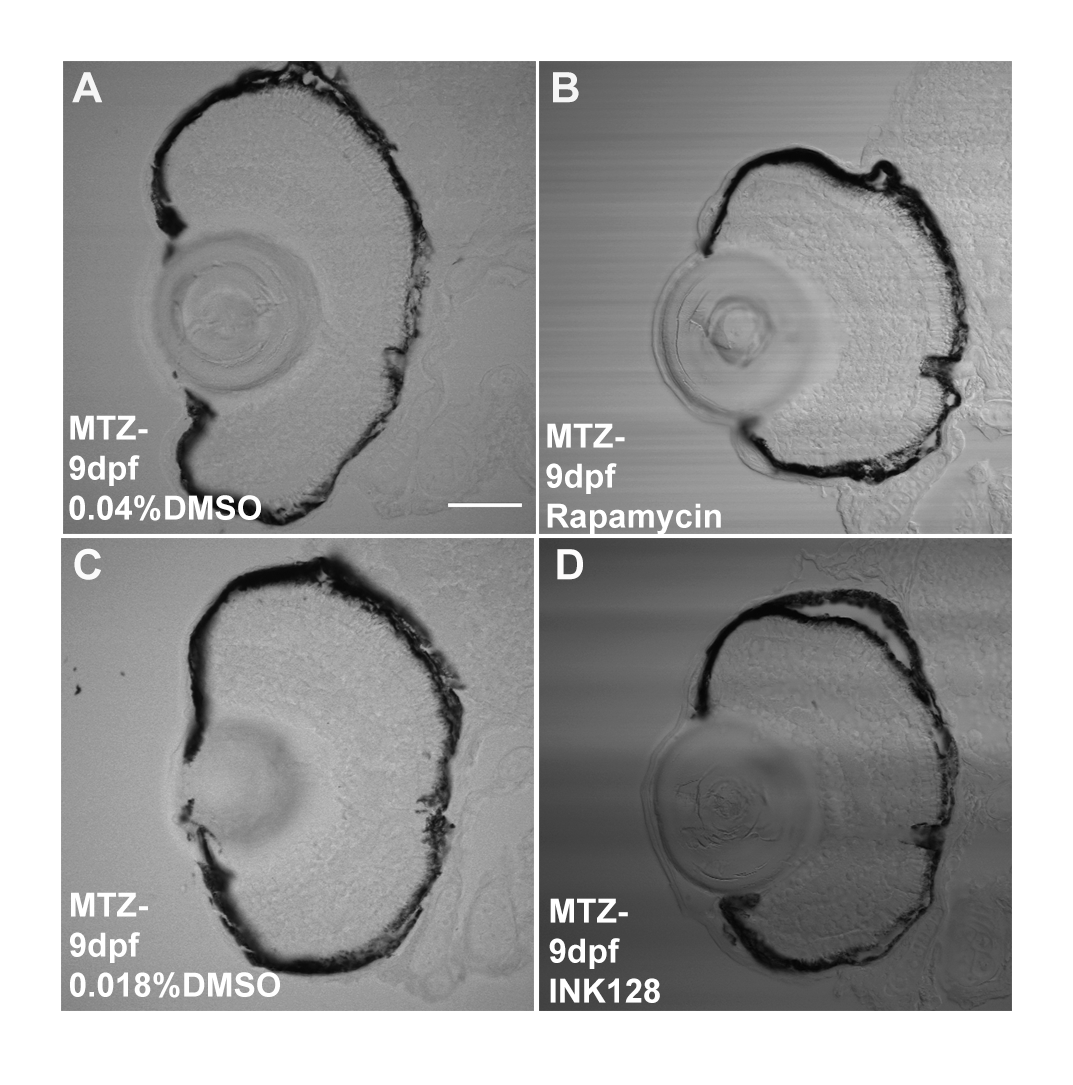

Supplement: S4 Fig — (A-D) Brightfield representative images of cryosections from MTZ- DMSO-, rapamycin- and INK128-treated larvae at 4dpi. Dorsal is up and distal is left. Scale bar = 50μm. (TIF) [file pgen.1009628.s004.tif]

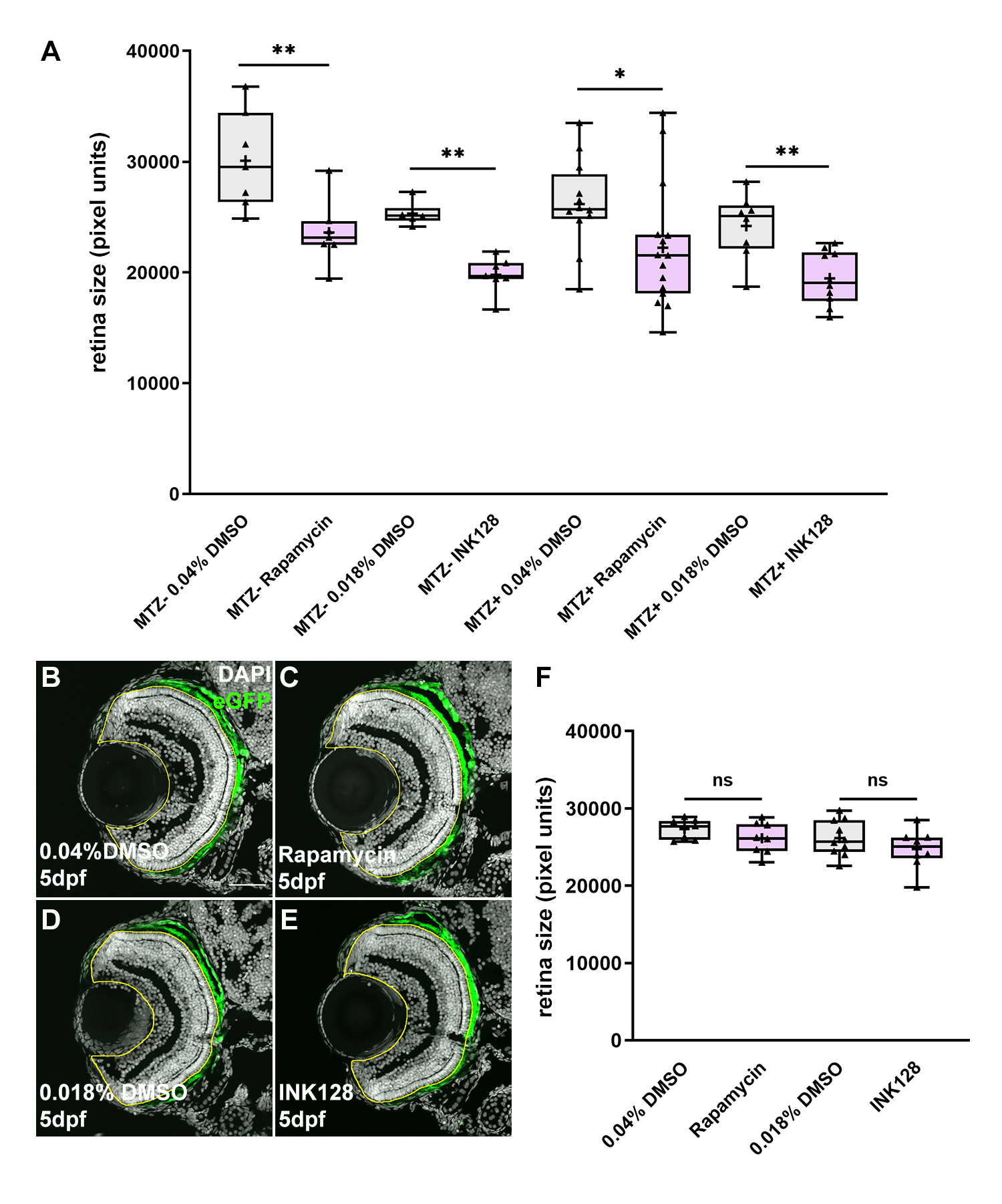

Supplement: S5 Fig — (A) Quantification of retinal size in larvae treated with DMSO, rapamycin, or INK128 from 4dpf-4dpi (long-term) showed significant decreases in inhibitor-treated larvae from unabated and ablated groups when compared to the corresponding DMSO-treated controls. (B-E) Fluorescent images on cryosections from DMSO- and rapamycin/INK128-treated larvae at 5dpf. Yellow lines outline the area of the neural retina for eye size measurement. (F) Quantification of retinal size of larvae treated with DMSO, rapamycin, or INK128 from 4dpf-5dpf (short-term) showed comparable overall size between inhibitor- and DMSO-treated groups. p-values: * ≤ 0.05, ** ≤ 0.01. Statistical information can be found in S9 Table. Nuclei (white), eGFP (green). Dorsal is up and distal is left. Scale bar = 50μm. (TIF) [file pgen.1009628.s005.tif]

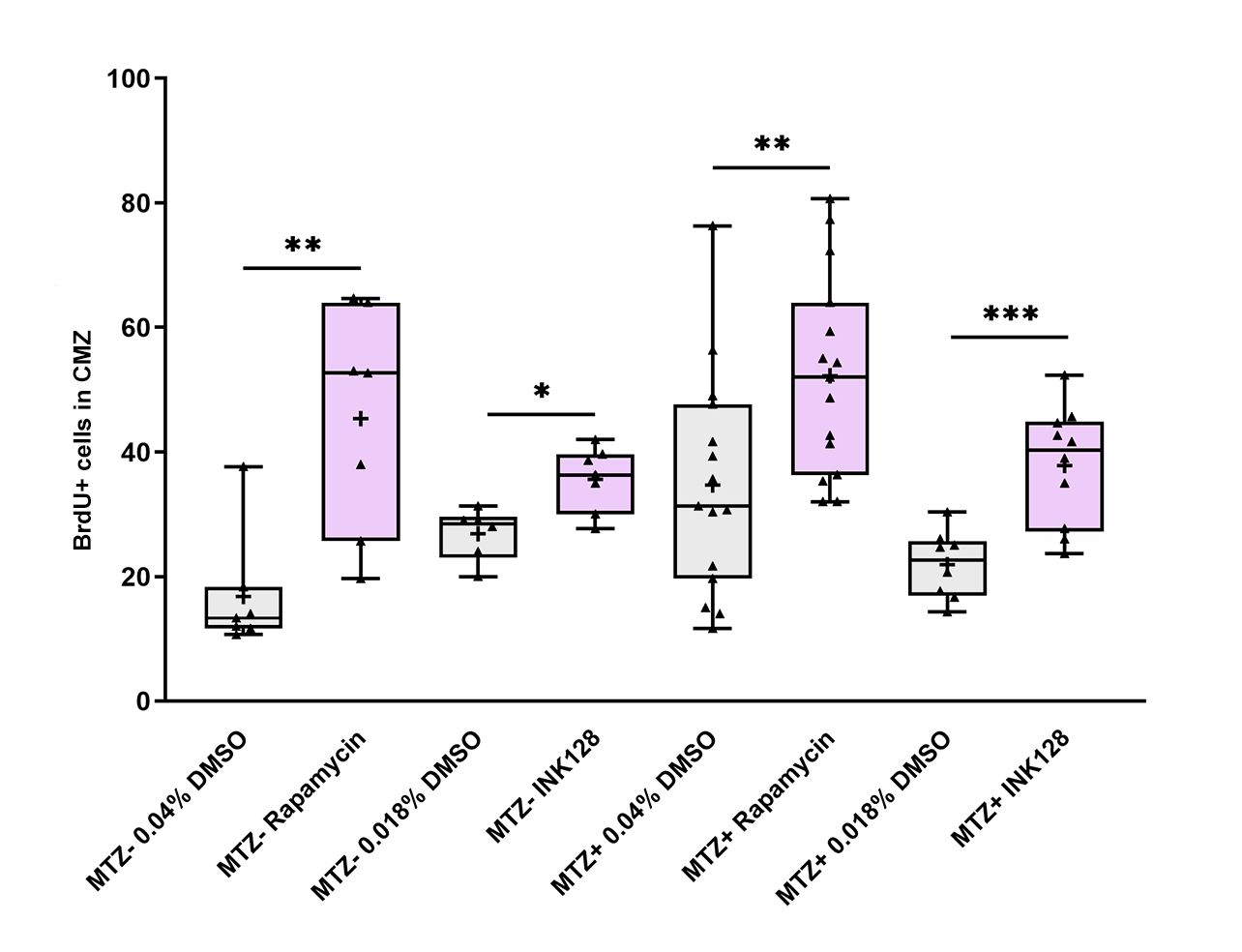

Supplement: S6 Fig — Quantification of BrdU+ cells in the ciliary marginal zone (CMZ) from larvae treated with DMSO, rapamycin, or INK128 from 4dpf - 4dpi showed significantly increased cell proliferation in mTOR inhibitor-treated larvae from unabated and ablated groups, compared with corresponding DMSO-treated controls. p-values: * ≤ 0.05, ** ≤ 0.01, and ***≤ 0.001. Statistical information can be found in S9 Table. (TIF) [file pgen.1009628.s006.tif]

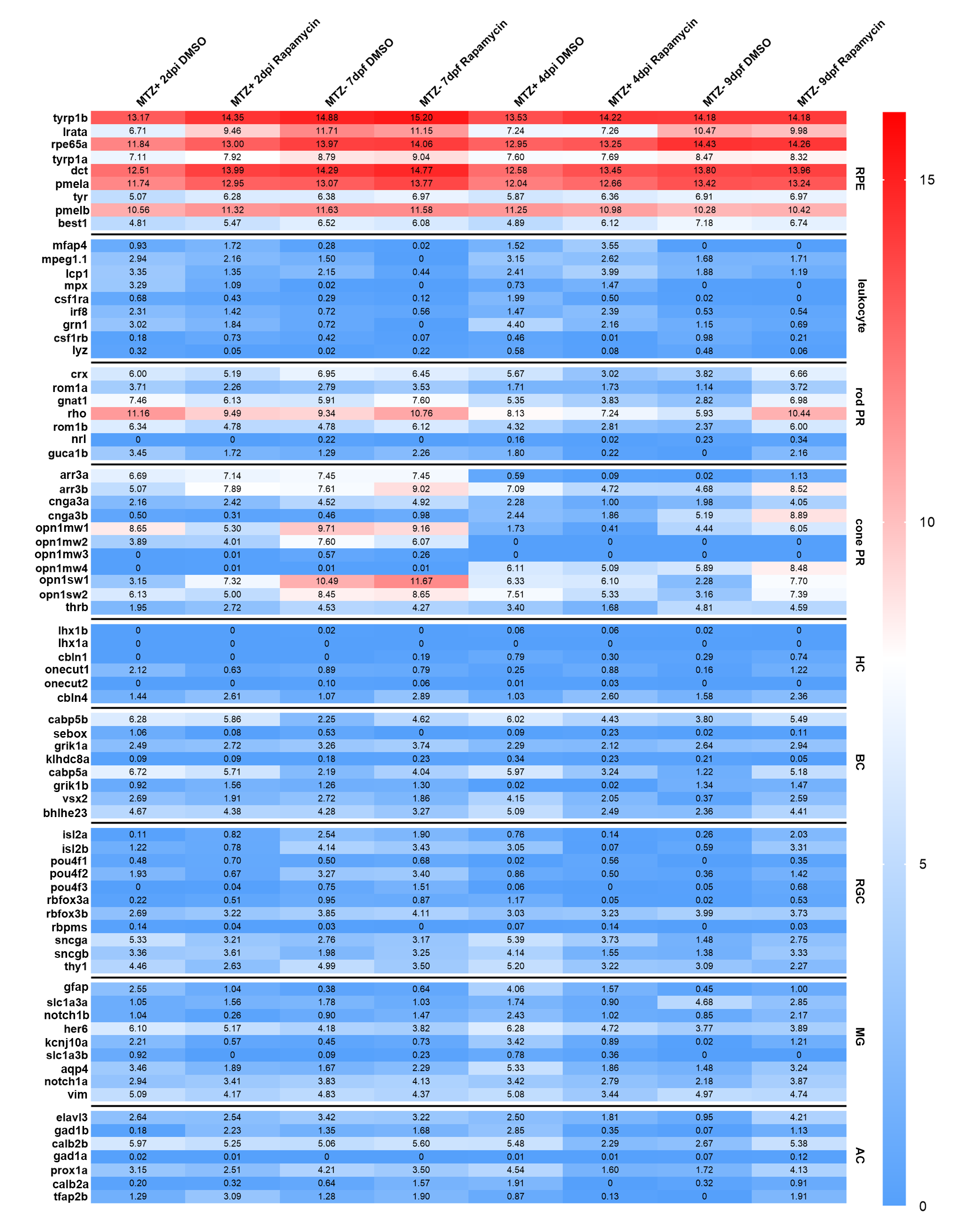

Supplement: S7 Fig — Heatmap showing average (n = 3) expression values of representative RPE, leukocyte, and retinal cell marker genes (including rod and cone photoreceptors (PR), bipolar cells (BC), retinal ganglion cells (RGC), amacrine (AC) cells and Muller glia (MG)) across the different treatment conditions examined by RNA-seq. The RPE markers (tyrp1a, lrata, rpe65a, tyrp1b, dct, pmela, tyr, pmelb, best1) show high expression across all 8 treatment groups relative to other cell type gene markers. The expression values of leukocyte and retinal cell marker genes appear relatively low, with the exception of some photoreceptor genes (rho, crx, arr3a, arr3b, opn1sw1, opn1sw2, opn1mw1, opn1mw2). Black lines separate marker gene groups for different cell types. Heatmap legend represents log2(counts per million mapped reads (CPM)+1). (TIF) [file pgen.1009628.s007.tif]

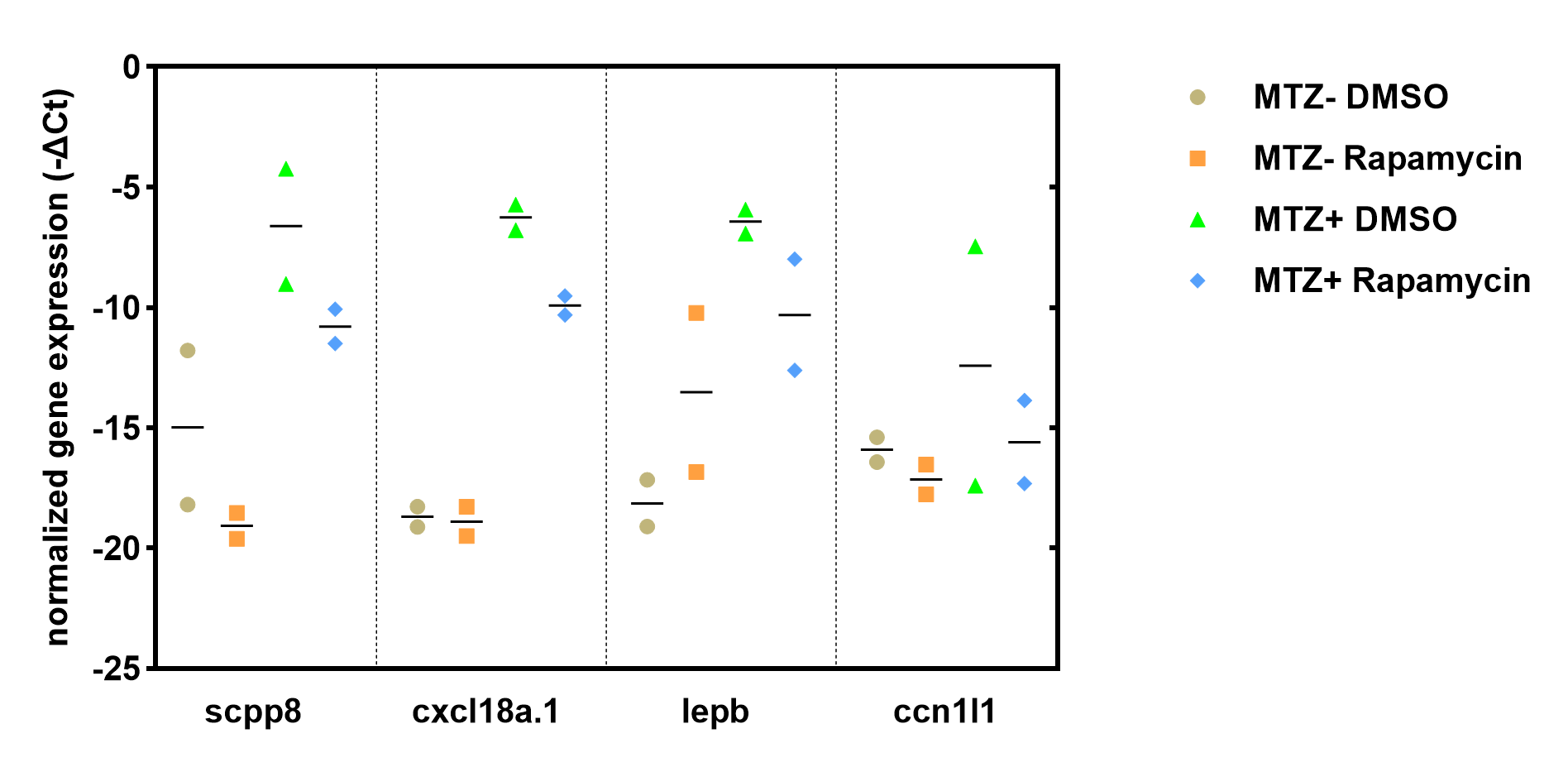

Supplement: S8 Fig — qRT-PCR validation of four differentially expressed genes (scpp8, cxcl18a.1, lepb, ccn1l1) in MTZ- DMSO, MTZ- Rapamycin, MTZ+ DMSO, and MTZ+ Rapamycin treatment groups used for RNA-seq at 2dpi/7dpf. Similar to RNA-seq results (see Fig 5D and S1 and S6 Tables), data show increased expression of all genes in MTZ+ DMSO groups (green) compared to MTZ- DMSO (tan) and MTZ+ Rapamycin groups (blue). Transcript levels were normalized to the expression of beta-actin. Lines represent the median. Each data point represents a biological replicate (n = 2). (TIF) [file pgen.1009628.s008.tif]
