# Supplementary material for: mTOR activity is essential for retinal pigment epithelium regeneration in zebrafish
Source: PLoS Genet. 2022 Mar 10;18(3):e1009628. doi: 10.1371/journal.pgen.1009628 (PMC8939802; doi:10.1371/journal.pgen.1009628)
Supplement: S1 Table — (PDF) [file pgen.1009628.s009.pdf]

S1 Table. MTZ[+] 2dpi rapamycin vs. dmso downregulated genes (top 100)

Gene name	Log_2 fold change	FDR p-value	Gene name	Log_2 fold change	FDR p-value
gna14	-8.85	0.05	aqp1a.1	-2.75	1.72E-03
ptf1a	-8.15	2.87E-04	apoeb	-2.75	7.54E-09
lrrc15	-7.22	2.67E-04	si:dkey-25e12.3	-2.65	0.02
scpp8	-6.11	0*	si:dkey-246e1.3	-2.63	0.03
igfbp5a	-5.82	1.63E-04	plekhn1	-2.63	6.07E-06
cyp1b1	-5.61	1.05E-11	stm	-2.62	1.32E-03
prdm13	-5.52	4.15E-03	lipib	-2.6	3.76E-04
si:ch211-66i15.5_1	-5.36	0.04	si:ch211-201h21.5	-2.59	0.02
aldoca	-5.32	0.04	rhbg	-2.57	5.66E-09
myod1	-4.76	0.02	st6gal2b	-2.55	0.01
saa	-4.55	0.01	slc38a4	-2.45	2.35E-05
bco2l	-4.47	0.04	cyp2ad6	-2.45	1.78E-04
ifitm1	-4.46	0.03	aanat1	-2.44	1.13E-06
mmp9	-4.3	8.25E-06	ddit4	-2.44	2.26E-04
g0s2	-4.29	4.25E-06	C25H11orf96	-2.43	0.04
cyp1d1	-4.26	9.95E-06	zgc:165604	-2.42	2.22E-07
nell2a	-4.08	1.40E-03	krt18a.1	-2.41	4.74E-03
tmx3a	-4.06	0.03	si:ch73-160h15.3	-2.41	1.10E-03
zgc:112285	-3.79	2.58E-05	fgf8b	-2.4	2.65E-03
si:ch211-270g19.5	-3.67	0.01	slc4a4a	-2.39	9.88E-04
cthl	-3.66	0.04	iqgap2	-2.37	9.33E-03
ccn1l1	-3.63	0.03	adma	-2.32	6.81E-03
cxcl18a.1	-3.63	2.62E-05	pygl	-2.31	1.25E-03
cyp2n13	-3.63	1.47E-04	nfasca	-2.31	1.97E-08
hspb6	-3.62	1.56E-03	eml2	-2.3	8.25E-06
lepb	-3.61	2.78E-04	sncga	-2.27	1.73E-03
inhbaa	-3.53	4.14E-03	timp2b	-2.26	8.90E-04
cpeb1a	-3.52	6.03E-03	rdh10a	-2.26	3.51E-04
irx5a	-3.48	1.41E-03	bgnb	-2.24	0.01
selenop	-3.38	0*	vill	-2.22	8.94E-04
aanat2	-3.31	0.02	slc13a1	-2.22	7.46E-03
ano5b	-3.27	3.84E-05	si:ch73-6k14.2	-2.15	0.02
si:ch73-52p7.1	-3.25	7.46E-03	CABZ01080074.1	-2.12	0.05
fgf8a	-3.2	9.33E-03	itgb5	-2.11	0.02
si:ch73-173h19.3	-3.17	0.02	sec14l8	-2.09	0.04
lrrn3a	-3.13	0.01	plcd1a	-2.09	0.02
ccdc80	-3.12	1.98E-03	krt8	-2.07	6.83E-04

foxi1	-3.12	9.44E-03	cacng7b	-2.06	0.03
fads2	-3.12	0.03	chst2b	-2.06	1.39E-03
cyp2p6	-3.1	5.69E-06	atp1b4	-2.03	1.54E-05
si:dkey-126g1.7	-3.04	0.05	CU929418.2	-2.03	1.32E-03
mpx	-3.02	3.97E-03	map1ab	-2.02	9.13E-04
serpinb1	-3.01	1.29E-09	il34	-1.97	8.14E-05
cyp46a1.2	-3	0.02	cadm4	-1.94	5.93E-03
si:dkey-23a13.2	-2.99	0.01	nexmifb	-1.94	0.03
cyp26a1	-2.94	2.31E-07	grm2b	-1.93	6.03E-03
fhl1a	-2.88	0.02	tubb5	-1.93	0.04
panx1b	-2.78	0.02	lmo7b	-1.93	8.10E-03
si:dkey-276j7.3	-2.77	0.01	ptgdsb.2	-1.91	1.00E-03
slc13a5a	-2.76	9.44E-03	atp1a3b	-1.89	0.02

Filters: Log2 fold change<-1; FDR p-value<0.05, Max group mean≥1

*: FDR p-value < 1E-16
