# Supplementary material for: mTOR activity is essential for retinal pigment epithelium regeneration in zebrafish
Source: PLoS Genet. 2022 Mar 10;18(3):e1009628. doi: 10.1371/journal.pgen.1009628 (PMC8939802; doi:10.1371/journal.pgen.1009628)
Supplement: S2 Table — (PDF) [file pgen.1009628.s010.pdf]

S2 Table. MTZ^{+} 2dpi rapamycin vs. dmso upregulated genes (top 100)

Gene name	Log_2 fold change	FDR p-value	Gene name	Log_2 fold change	FDR p-value
zgc:136930	11.73	3.90E-03	shisa2a	1.91	2.69E-03
hbbe1.3	11	0.02	hook1	1.87	9.33E-03
hbae3	8.43	1.79E-03	gdf6a	1.87	0.01
krt91	8.37	0*	si:ch211-51h9.7	1.86	0.01
krt4	7.45	0*	CU651662.1	1.85	5.87E-04
ascl1b_2	6.8	0.03	si:ch211-175m2.5	1.84	6.75E-03
krtt1c19e	6.79	1.37E-11	zgc:195173	1.84	0.03
cyt1	6.44	1.38E-09	tmem72	1.76	0.02
krt5	6.43	1.05E-11	slc27a1b_2	1.69	0.02
lgals1l1	6.12	3.69E-03	zgc:112148	1.69	0.05
cyt1l	5.75	2.92E-03	nsa2	1.68	0.01
dnase1l4.1	5.32	9.44E-03	elp4	1.67	0.02
si:rp71-77l1.1	4.98	2.82E-03	ahcy	1.67	8.10E-03
krt97	4.94	0*	rab38c	1.67	0.01
prkn	4.83	0.01	fabp11b	1.67	0.03
tgfbi	4.83	1.54E-03	si:ch211-251b21.1	1.66	0.01
zgc:92380	4.66	1.80E-03	spink2.1	1.65	0.01
zgc:92360	4.3	0.01	dmac2l	1.64	0.01
opn1sw1	4.3	1.68E-03	alas1	1.63	2.21E-03
cxl34b.11	4.12	1.46E-03	sod3a	1.61	0.03
zgc:101810	4.12	0.03	pcmtl	1.61	0.01
eno4	4	0.04	gstr	1.61	2.07E-03
col1a1b	3.76	3.69E-03	pfn1	1.6	0.02
ctsl.1	3.52	0.02	eef2l2	1.58	0.01
necab1	3.26	0.02	ino80c	1.58	0.02
si:ch211-113a14.12	3.19	0.01	bloc1s1	1.58	0.02
tmsb1	3.15	5.14E-08	cd151	1.57	7.08E-03
si:dkey-40g16.6	3.05	3.90E-03	il10rb	1.56	9.88E-03
gpr78a	2.8	0.02	hikeshi	1.55	8.56E-03
pcmtd2a	2.77	0.03	unkl	1.55	0.02
lrata	2.73	1.82E-03	mterf3	1.55	0.01
masp1	2.65	7.75E-04	fkbp1b	1.55	0.01
zgc:73226	2.57	1.55E-03	romo1	1.54	0.03
tspan4b	2.55	5.77E-04	plin1	1.51	0.02
sfrp1a	2.5	2.03E-03	tm6sf2	1.5	0.02
si:ch211-196i2.1	2.48	0.03	itm2bb	1.48	0.02

eef1da	2.33	1.48E-06	ccni	1.48	0.03
klhl24b	2.32	0.01	zgc:158482	1.48	0.01
gapdh	2.31	0.01	dct	1.47	0.02
tcn2	2.28	7.13E-07	caprin1b	1.47	3.69E-03
hyi	2.25	2.87E-04	ddx21	1.47	0.02
aldob	2.22	2.87E-05	mbd4	1.47	0.04
si:ch1073-406l10.2	2.2	3.16E-03	pno1	1.47	0.02
CABZ01090041.1	2.01	1.55E-03	ngrn	1.45	0.03
si:dkey-85k7.7	2.01	9.84E-04	tpt1	1.45	0.02
sfrp5	2	8.32E-03	eif4ebp3	1.44	0.02
si:ch211-161c3.6	2	0.03	zgc:92242	1.44	0.03
rab3db	1.98	3.20E-03	tbc1d15	1.44	0.03
myh9a	1.92	0.03	rfxap	1.41	0.04
nudt12	1.91	0.04	mtus1a	1.4	0.02

Filters: Log2 fold change>1; FDR p-value<0.05, Max group mean≥1

*: FDR p-value < 1E-16
