# Supplementary material for: mTOR activity is essential for retinal pigment epithelium regeneration in zebrafish
Source: PLoS Genet. 2022 Mar 10;18(3):e1009628. doi: 10.1371/journal.pgen.1009628 (PMC8939802; doi:10.1371/journal.pgen.1009628)
Supplement: S3 Table — (PDF) [file pgen.1009628.s011.pdf]

S3 Table. MTZ[+] 4dpi rapamycin vs. dmso downregulated genes (top 100)

Gene name	Log_2 fold change	FDR p-value	Gene name	Log_2 fold change	FDR p-value
asic2	-10.44	7.18E-04	tmem244	-5.06	7.04E-05
si:dkey-196h17.9	-10.27	1.03E-03	pdk3b	-5.06	7.01E-06
cfd	-9.57	7.28E-03	kcng4b	-5.04	4.28E-06
calb2a	-9.3	0.01	si:ch211-256a21.4	-4.97	4.00E-02
dtnbp1b	-9.23	6.15E-03	opn4a	-4.95	1.00E-02
krt94	-9.21	1.00E-02	IGLON5	-4.89	8.33E-03
esama	-9.19	7.83E-03	aqp1a.1	-4.87	0.00E+00*
clec14a	-9.08	0.02	CABZ01071020.1	-4.87	1.56E-03
calcrla	-8.91	0.01	nfixb	-4.87	2.73E-11
igf1	-8.82	0.02	her4.1	-4.84	0.00E+00
impdh1a	-8.54	7.67E-07	apela	-4.83	1.00E-02
rims2a	-8.37	5.60E-06	cryba4	-4.8	5.85E-03
rbp7a	-8.29	0.04	rnf182	-4.8	1.82E-13
si:dkey-33i11.9	-8.24	4.00E-02	LO018231.1	-4.71	4.00E-11
si:dkey-219c10.4	-7.89	1.68E-08	her4.2_2	-4.7	6.71E-11
igfbp1b	-7.84	4.73E-08	gabrr3b	-4.7	1.38E-03
gfi1ab	-7.73	4.00E-03	CABZ01114053.1	-4.66	8.59E-05
her2	-7.32	2.46E-03	phf24	-4.66	1.41E-07
ccl34b.1	-7.28	6.10E-11	si:ch211-125c23.3	-4.61	1.59E-04
slc43a3a	-7.28	2.71E-04	SLC6A13	-4.6	5.47E-03
si:ch211-127b11.1	-7.24	4.91E-06	zgc:136858	-4.59	3.66E-05
krt98	-7.24	5.97E-04	cyp2n13	-4.55	0.00E+00*
bco2l	-7.07	1.69E-04	stxbp6l	-4.53	0.00E+00*
isl2b	-7.03	4.14E-06	CR847844.1	-4.51	3.72E-03
si:ch211-191i18.4	-6.82	9.42E-05	calcr	-4.51	2.23E-05
gpsm1a	-6.59	4.39E-13	gad1b	-4.51	1.21E-04
gbx2	-6.37	1.77E-04	prss56	-4.5	2.00E-02
desma	-6.32	1.87E-03	si:ch211-37e10.2	-4.5	5.47E-06
rasd2	-6.19	1.76E-06	kiss2	-4.5	3.22E-03
si:ch211-165b10.3	-6.12	1.99E-03	her4.2_1	-4.49	6.52E-11
slc6a1a	-6.05	3.68E-09	slc6a1b	-4.48	9.28E-05
cart4	-6.02	2.54E-03	tmeff2a	-4.47	1.98E-03
cd83	-5.95	0.00E+00*	mical3b	-4.47	3.44E-05
samd10a	-5.91	4.00E-02	cdc42ep1b	-4.45	2.00E-02
ccdc151	-5.9	1.50E-03	myo15aa	-4.37	0.00E+00*
ucp1	-5.71	0.00E+00*	stmn4	-4.34	1.53E-03
FNDC10	-5.67	3.81E-04	bmp1b	-4.33	2.90E-11

her12	-5.61	0.00E+00*	rhol	-4.31	2.00E-02
si:ch211-270g19.5	-5.56	0.00E+00*	calml4b	-4.3	5.39E-06
si:dkey-76d14.2	-5.55	2.00E-03	cnksr2b	-4.29	0.00E+00*
adcyap1r1a_2	-5.54	0.00E+00*	inka1b	-4.29	1.00E-02
cela1.4	-5.53	4.12E-03	si:ch211-214p13.3	-4.28	0.00E+00*
si:dkeyp-44b5.4_1	-5.5	7.15E-03	isl1	-4.28	8.87E-12
si:dkey-11k2.7	-5.45	9.63E-03	fgf8b	-4.26	1.35E-08
FP016205.1	-5.39	3.00E-02	cyp26c1	-4.25	5.76E-12
tyrobp	-5.38	4.00E-02	agap3	-4.25	2.53E-05
cx31.7	-5.2	0.00E+00*	selenop	-4.25	0.00E+00*
flrt2_1	-5.2	4.30E-08	rergla	-4.25	3.70E-07
cyp4t8	-5.15	3.20E-12	spock1	-4.24	2.93E-03
atp6ap1lb	-5.09	2.04E-10	hey1	-4.23	1.64E-08

Filters: Log2 fold change<-1; FDR p-value<0.05, Max group mean≥1

*: FDR p-value < 1E-16
