# Supplementary material for: mTOR activity is essential for retinal pigment epithelium regeneration in zebrafish
Source: PLoS Genet. 2022 Mar 10;18(3):e1009628. doi: 10.1371/journal.pgen.1009628 (PMC8939802; doi:10.1371/journal.pgen.1009628)
Supplement: S4 Table — (PDF) [file pgen.1009628.s012.pdf]

S4 Table. MTZ+ 4dpi rapamycin vs. downregulated genes (top 100)

Gene name	Log₂ fold change	FDR p-value	Gene name	Log₂ fold change	FDR p-value
zgc:173915_2	10.66	5.69E-03	prmt3	1.66	1.50E-03
si:ch211-194m7.3	9.59	0.03	utp20	1.66	0.01
spp1	9.45	0.03	ddx49	1.66	3.71E-03
ebi3	4.3	1.11E-03	slc25a40	1.65	4.72E-03
acta1a	4.1	3.84E-05	angptl4	1.65	3.70E-03
dkk1a	3.82	2.57E-05	twistnb	1.64	4.23E-03
zgc:86709	3.61	0.02	cdh1	1.63	1.59E-03
zgc:66313	3.46	1.73E-08	map2k1	1.61	1.55E-03
nppb	3.4	0.03	dtwd1	1.59	9.75E-03
sema3gb	3.17	9.69E-03	gnl1	1.58	3.70E-03
noxo1b	2.9	2.60E-07	mphosph10	1.58	4.65E-03
cpt1b	2.64	7.37E-03	urb2	1.58	0.02
rgs5b	2.62	5.43E-04	bmf2	1.58	0.01
ptgs2a	2.5	3.89E-03	tpt1	1.57	1.78E-03
il11b	2.42	0.01	pim1	1.57	1.99E-03
polr3gla	2.39	6.41E-05	si:ch73-335l21.4	1.57	1.49E-03
hp_2	2.38	8.82E-03	c19h1orf109	1.56	0.03
f3b	2.37	1.94E-04	elp4	1.56	0.02
ccn1	2.32	3.83E-05	cyp1a	1.56	0.03
si:dkey-184p18.2	2.28	6.75E-05	nup54	1.55	1.58E-03
tubb6	2.24	5.52E-04	tpk2	1.55	0.02
mlf1	2.24	1.14E-03	MTERF4	1.54	0.02
twnk	2.23	1.50E-03	bms1	1.54	0.01
cpt1ab	2.21	4.05E-05	tsr1	1.53	7.37E-03
ppm1h	2.18	6.94E-04	rbms1a	1.53	0.04
hspa8b	2.04	2.10E-03	noc2l	1.53	0.01
ddx21	2.03	5.76E-06	CU651662.1	1.53	0.02
PSTK	1.92	3.14E-03	mafk	1.52	0.02
ocstamp	1.92	4.68E-04	tmem88b	1.52	0.03
hsp70.1	1.92	4.94E-03	slc38a2	1.52	1.72E-03
cxcl12b	1.91	0.02	xpnpep1	1.51	2.12E-03
slc43a1a	1.84	9.42E-03	npr3	1.51	9.91E-04
tspan4b	1.83	0.01	oplah	1.51	0.02
si:dkey-48p11.3	1.83	5.76E-03	tsen15	1.51	0.04
hsp70.2	1.81	2.43E-03	cnn2	1.5	3.12E-03
slc5a3b	1.81	2.15E-04	msh2	1.5	0.03
si:dkey-79d12.5	1.81	1.94E-04	hsp70.3	1.5	0.01

dph6	1.79	3.63E-03	zcchc9	1.5	3.61E-03
gtpbp4	1.79	1.45E-04	lmna	1.5	0.02
zgc:100920	1.78	4.65E-03	csrp1a	1.49	3.32E-03
klhl24a	1.78	0.05	si:dkey-184p9.7	1.49	0.02
lyar	1.78	6.52E-04	znhit6	1.49	0.04
dph1	1.77	3.06E-03	f3a	1.48	4.11E-03
cntfr	1.76	4.44E-04	gask1b	1.48	0.03
nufip1	1.74	3.11E-03	zgc:77158	1.48	0.01
fosl1a	1.73	0.01	lhfpl6	1.48	5.16E-03
tpcn2	1.73	0.04	sh3bp4a	1.48	0.03
pno1	1.71	4.44E-04	hif1an	1.48	0.05
sts	1.7	0.05	ACOT12	1.47	0.03
slc27a1b_2	1.67	0.01	nudt1	1.46	0.02

Filters: Log2 fold change>1; FDR p-value<0.05, Max group mean≥1
