# Supplementary material for: mTOR activity is essential for retinal pigment epithelium regeneration in zebrafish
Source: PLoS Genet. 2022 Mar 10;18(3):e1009628. doi: 10.1371/journal.pgen.1009628 (PMC8939802; doi:10.1371/journal.pgen.1009628)
Supplement: S5 Table — (PDF) [file pgen.1009628.s013.pdf]

S5 Table. MTZ$^+$ 2dpi rapamycin vs. DMSO enriched downregulated reactome pathways

pathway ID	pathway description	gene count	FDR p-value	matching genes
DRE-382551	Transport of small molecules	14	0.0046	clic2,atp1a1b,aqp1a.1,slc4a4a,slc13a1,rhbg,apoeb,atp1b3b,slc38a4,atp1a3a,slc13a5a,ano5b,slc1a2b,si:ch211-270g19.5
DRE-168256	Immune System	15	0.0437	pygl,mpx,tubb5,qsox1,mmp9,serpinb1,itgb5,krt8,panx1b,timp2b,nfasca,clu,il34,iqgap2,cxcl18a.1
DRE-168249	Innate Immune System	13	0.0352	pygl,mpx,tubb5,qsox1,mmp9,serpinb1,krt8,panx1b,timp2b,nfasca,clu,iqgap2,cxcl18a.1
DRE-6798695	Neutrophil degranulation	10	0.0098	pygl,mpx,tubb5,qsox1,mmp9,serpinb1,krt8,timp2b,nfasca,iqgap2
DRE-397014	Muscle contraction	7	0.0098	clic2,atp1a1b,atp1b3b,itgb5,atp1a3a,cacng7b,ENSDARG00000097256
DRE-1474244	Extracellular matrix organization	7	0.035	bgnb,mmp9,serpine1,itgb5,timp2b,LOC563864,col15a1b
DRE-5576891	Cardiac conduction	6	0.0098	clic2,atp1a1b,atp1b3b,atp1a3a,cacng7b,si:ch211-270g19.5
DRE-983712	Ion channel transport	6	0.0098	clic2,atp1a1b,atp1b3b,atp1a3a,ano5b,si:ch211-270g19.5
DRE-425407	SLC-mediated transmembrane transport	6	0.035	slc4a4a,slc13a1,rhbg,slc38a4,slc13a5a,slc1a2b
DRE-5578775	Ion homeostasis	5	0.0046	clic2,atp1a1b,atp1b3b,atp1a3a,si:ch211-270g19.5

Filters: gene counts ≥5, FDR p-value<0.05
