# Supplementary material for: mTOR activity is essential for retinal pigment epithelium regeneration in zebrafish
Source: PLoS Genet. 2022 Mar 10;18(3):e1009628. doi: 10.1371/journal.pgen.1009628 (PMC8939802; doi:10.1371/journal.pgen.1009628)
Supplement: S6 Table — (PDF) [file pgen.1009628.s014.pdf]

S6 Table. MTZ⁺ 2dpi DMSO vs. 7dpf MTZ- DMSO upregulated genes (top 100)

Gene name	Log_2 fold change	FDR p-value	Gene name	Log_2 fold change	FDR p-value
mpz	13.83	5.79E-04	si:dkey-276j7.3	5.27	5.40E-03
il11b	13.05	4.67E-04	si:dkey-23a13.2	5.16	3.29E-03
ptgs2b	12.68	7.89E-04	si:ch1073-385f13.3	5.15	0.01
plekhn1	12.1	1.69E-03	igfbp5a	5.13	0.04
lepb	11.42	3.59E-03	ahsa1a	5	0.04
ccn1l1	10.48	0.01	sall1b	4.96	3.91E-03
cxcl18a.1	10.47	0.01	cyp26a1	4.92	1.60E-04
CABZ01081490.1	10.07	0.02	lifrb	4.81	1.04E-03
tcap	9.83	0.03	zte38	4.66	0.04
cldn7b	9.78	0.03	si:dkey-184p18.2	4.66	2.18E-04
AL935186.1	9.7	0.03	atf3	4.63	1.00E-03
krt18a.1	9.64	9.23E-08	casq2	4.6	2.96E-03
cpeb1a	9.48	0.04	si:ch1073-170o4.1	4.59	0.01
plekhs1	9.16	2.41E-04	fhl1a	4.56	3.34E-03
il11a	8.92	2.40E-04	il34	4.56	4.58E-04
cnga2b	8.69	6.83E-04	arg2	4.52	1.60E-04
mpx	8.31	1.41E-03	cacng7b	4.49	0.01
cdab	7.89	3.59E-03	itgb3b	4.49	0.01
si:ch211-153b23.5	7.89	3.47E-05	tagln2	4.26	0.04
cidec	7.88	6.76E-06	smyd5	4.16	1.81E-03
adamtsl7	7.87	2.67E-05	rbms1a	4.13	9.72E-04
epcam	7.87	6.13E-06	sat1a.2	4.12	5.79E-04
serpine1	7.82	1.60E-04	wnt11f2	4.1	0.05
ptgs2a	7.71	6.76E-06	ets2	4.1	7.37E-03
cxcl8a	7.71	7.89E-04	afap1l1b	4.08	0.02
tmod4	7.5	1.29E-04	fthl28	4.08	1.69E-03
si:ch73-56d11.5	7.13	1.14E-03	si:dkey-126g1.7	4.05	0.05
adma	6.95	2.56E-04	flj13639	4.01	0.01
edn2	6.88	3.12E-05	pprc1	4.01	2.65E-03
clcf1	6.53	1.62E-04	pnp5b	3.99	9.62E-03
myod1	6.52	0.03	cbsa	3.97	6.18E-03
zgc:153911	6.5	2.37E-04	cabp5a	3.96	7.20E-03
scpp8	6.45	1.60E-04	hsd17b12a	3.94	6.54E-03
fosl1b	6.43	1.29E-04	per2	3.93	5.99E-03
nfe2l2b	6.36	5.71E-03	si:busm1-57f23.1	3.93	2.13E-03
cfl1l	6.33	1.29E-04	fthl27_2	3.9	4.26E-03
CABZ01086293.1	6.31	0.02	cdh1	3.88	1.73E-03

mmp9	6.26	1.60E-04	dennd2da	3.87	0.02
lgsn	6.15	2.01E-03	akap12b	3.81	2.40E-03
ptx3a	6.14	3.52E-03	igfbp1a	3.78	3.59E-03
epha2a	6.09	1.73E-03	dkk1a	3.77	0.01
met	5.82	3.57E-03	fosl1a	3.77	5.25E-03
cpa4	5.64	0.03	apoeb	3.77	2.14E-03
si:dkey-23i12.7	5.6	2.92E-03	cxcl18b	3.76	6.08E-03
notum1b	5.58	5.89E-03	e2f7	3.75	4.26E-03
slc38a8b	5.54	9.39E-03	ocstamp	3.75	7.41E-03
foxi1	5.49	0.01	npm1a	3.68	3.22E-03
mmp13b	5.4	2.14E-03	hk2	3.67	5.25E-03
zgc:112285	5.3	0.02	si:ch1073-303k11.2	3.65	9.39E-03
si:ch211-204c21.1_1	5.28	0.02	hsph1	3.64	0.02

Filters: Log2 fold change>1; FDR p-value<0.05, Max group mean≥1
