# Supplementary material for: mTOR activity is essential for retinal pigment epithelium regeneration in zebrafish
Source: PLoS Genet. 2022 Mar 10;18(3):e1009628. doi: 10.1371/journal.pgen.1009628 (PMC8939802; doi:10.1371/journal.pgen.1009628)
Supplement: S7 Table — (PDF) [file pgen.1009628.s015.pdf]

S7 Table. MTZ$^+$ 4dpi rapamycin vs. DMSO enriched downregulated reactome pathways (top 10)

pathway ID	pathway description	gene count	FDR p-value	matching genes
DRE-112315	Transmission across Chemical Synapses	23	0.00011	gabrr1,slc6a1b,gnb3b,slc18a3a,gnb3a,lin7a,grin1b,gng13b,glrbb,vamp2,gabrb3,gabrg2,SLC6A13,glrba,nsfa,kcnj10a,adcy7,slc6a1a,gad1b,gngt2a,camk2b1,abat,gabrr3b
DRE-112316	Neuronal System	28	0.0015	gabrr1,slc6a1b,gnb3b,slc18a3a,gnb3a,lin7a,grin1b,gng13b,LOC566228,glrbb,vamp2,gabrb3,gabrg2,SLC6A13,abcc8b,homer3,glrba,nsfa,kcnj10a,adcy7,slc6a1a,gad1b,kcnv2a,gngt2a,hcn2b,camk2b1,abat,gabrr3b
DRE-112310	Neurotransmitter release cycle	8	0.0039	slc6a1b,slc18a3a,lin7a,vamp2,SLC6A13,slc6a1a,gad1b,abat
DRE-382551	Transport of small molecules	37	0.0039	slc6a1b,gnb3b,ldlrap1a,atp1b3a,gnb3a,atp1a1b,aqp4,cahz,aqp1a.1,add3a,slc4a4a,ldlr,slc3a2a,gng13b,rhbg,apoeb,bmp1b,atp1b3b,slc38a4,slc7a10a,slc47a2,slc12a7b,abcg1,SLC6A13,atp1b2b,slc25a18,atp2b1b,atp1a3a,slc13a5a,adcy7,slc6a1a,slc43a2b,gngt2a,camk2b1,stom,slc2a3a,ENSDARG00000097256
DRE-4086398	Ca2+ pathway	8	0.0039	pde6a,gnb3b,gnb3a,fzd5,gnao1b,gng13b,gnao1a,gngt2a
DRE-418594	G alpha (i) signalling events	22	0.0039	pde6a,gnb3b,grm7,bco2l,gnb3a,rlbp1a,penkb,gnao1b,cxcl12a,gng13b,cxcr4b,gpr37l1b,rcvrna,ppp1r1b,adora1a,gpr37a,adcy7,gnao1a,ENSDARG00000055722,saga,gngt2a,sagb
DRE-888590	GABA synthesis, release, reuptake and degradation	5	0.0039	slc6a1b,SLC6A13,slc6a1a,gad1b,abat
DRE-112314	Neurotransmitter receptors and postsynaptic signal transmission	15	0.0042	gabrr1,gnb3b,gnb3a,grin1b,gng13b,glrbb,gabrb3,gabrg2,glrba,nsfa,kcnj10a,adcy7,gngt2a,camk2b1,gabrr3b
DRE-936837	Ion transport by P-type ATPases	8	0.0043	atp1b3a,atp1a1b,atp1b3b,atp1b2b,atp2b1b,atp1a3a,camk2b1,ENSDARG00000097256

DRE-977443	GABA receptor activation	9	0.00011	gabrr1,slc6a1b,gnb3b,slc18a3a,gnb3a,lin7a,grin1b,gng13b,glrbb,vamp2,gabrb3,gabrg2,SLC6A13,glrba,nsfa,kcnj10a,adcy7,slc6a1a,gad1b,gngt2a,camk2b1,abat,gabrr3b

Filters: gene counts ≥5, FDR p-value<0.05
