# Supplementary material for: mTOR activity is essential for retinal pigment epithelium regeneration in zebrafish
Source: PLoS Genet. 2022 Mar 10;18(3):e1009628. doi: 10.1371/journal.pgen.1009628 (PMC8939802; doi:10.1371/journal.pgen.1009628)
Supplement: S8 Table — (PDF) [file pgen.1009628.s016.pdf]

S8 Table. MTZ$^+$ 2dpi DMSO vs. 7dpf MTZ$^-$DMSO (*csf1ra, csf1rb*)

Timepoints	Gene name	Log_2 fold change	FDR p-value	Average CPM	
				MTZ$^-$ DMSO	MTZ$^+$ DMSO
2dpi/7dpf	*csf1ra*	0.11	1.00	17.0	17.6
	csf1rb	-0.41	1.00	3.33	9.0
4dpi/9dpf	*csf1ra*	6.39	0.04	82.3	0.3
	csf1rb	-0.55	0.98	8.3	27

CPM: counts per million
