# Supplementary material for: mTOR activity is essential for retinal pigment epithelium regeneration in zebrafish
Source: PLoS Genet. 2022 Mar 10;18(3):e1009628. doi: 10.1371/journal.pgen.1009628 (PMC8939802; doi:10.1371/journal.pgen.1009628)
Supplement: S9 Table — (PDF) [file pgen.1009628.s017.pdf]

S9 Table. Statistics

Figures	Numbers of independent experiments (N)	Compared groups	Statistical tests	Biological replicates (n)	p-values
Fig 1O	N=1	3hpi MTZ^- ,3hpi MTZ^+	Mann-Whitney test	n=8, n=10	p=0.6965
	N=2	6hpi MTZ^-, 6hpi MTZ^+	Mann-Whitney test	n=10, n=10	p=0.0003
	N=1	12hpi MTZ^-, 12hpi MTZ^+	Mann-Whitney test	n=7, n=9	p=0.0052
	N=2	1dpi MTZ^-, 1dpi MTZ^+	Mann-Whitney test	n=5, n=8	p=0.0016
	N=2	2dpi MTZ^-, 2dpi MTZ^+	Mann-Whitney test	n=6, n=9	p=0.0004
	N=2	3dpi MTZ^-, 3dpi MTZ^+	Mann-Whitney test	n=5, n=8	p=0.0016
	N=2	4dpi MTZ^-, 4dpi MTZ^+	Mann-Whitney test	n=9, n=7	p=0.4634
Fig 2E	N=2	0.04% DMSO, 2μM rapamycin	Mann-Whitney test	n=8, n=6	p=0.0007
	N=1	0.018% DMSO, 0.9μM INK128	Mann-Whitney test	n=6, n=6	p=0.0022
Fig 2K	N=3	0.04% DMSO, 2μM rapamycin	Mann-Whitney test	n=7, n=7	p>0.9999
	N=2	0.018%DMSO, 0.9μM INK128	Mann-Whitney test	n=6, n=7	p=0.4371
Fig 2P	N=3	0.04% DMSO, 2μM rapamycin	unpaired student t-test with Welch's correction	n=12, n=15	p=0.0006
	N=2	0.018%DMSO, 0.9μM INK128	Mann-Whitney test	n=8, n=10	p<0.0001
Fig 2U	N=3	0.04% DMSO, 2μM rapamycin	Mann-Whitney test	n=12, n=15	p<0.0001
	N=2	0.018% DMSO, 0.9μM INK128	unpaired student t-test with Welch's correction	n=8, n=10	p<0.0001
	N=1	0.018% DMSO, 0.9μM INK128	Mann-Whitney test	n=8, n=5	p=0.1810
Fig 3C	N=1	$mtor^{+/+}$, $mtor^{-/-}$	Mann-Whitney test	n=7, n=8	p=0.0205
Fig 3H	N=2	$MTZ^+ mtor^{+/+}$, MTZ^+ $mtor^{-/-}$	Mann-Whitney test	n=7, n=7	p=0.0105
	N=2	$MTZ^- mtor^{+/+}$, MTZ- $mtor^{-/-}$	Mann-Whitney test	n=6, n=7	p=0.1923
Fig 3K	N=3	$MTZ^+ mtor^{+/+}$, MTZ^+ $mtor^{-/-}$	unpaired student t-test with Welch's correction	n=9, n=9	p=0.0083
Fig 4R	N=2	MTZ^+ 2dpi 0.04% DMSO, MTZ^+ 2dpi 2μM MHY1485	Mann-Whitney test	n=8, n=9	p=0.0360

Fig 4S	N=2	8dpf MTZ^- 0.04% DMSO, 8dpf MTZ^- 2µM MHY1485	Mann-Whitney test	n=9, n=7	p=0.5631
	N=2	3dpi MTZ^+ 0.04% DMSO, 3dpi MTZ^+ 2µM MHY1485	unpaired student t-test with Welch's correction	n=10, n=10	p<0.0001
	N=1	7dpf MTZ^- 0.04% DMSO, 7dpf MTZ^- 2µM MHY1485	Mann-Whitney test	n=6, n=7	p=0.5973
	N=1	2dpi MTZ^+ 0.04% DMSO, 2dpi MTZ^+ 2µM MHY1485	unpaired student t-test with Welch's correction	n=8, n=8	p=0.8839
	N=1	9dpf MTZ- 0.04% DMSO, 9dpf MTZ+ 0.04% DMSO	Mann-Whitney test	n=6, n=8	p=0.0759
	N=1	4dpi MTZ^+ 0.04% DMSO, 4dpi MTZ^+ 2µM MHY1485	Mann-Whitney test	n=7, n=8	p=0.8048
Fig 4T	N=3	MTZ^+ 3dpi 0.04% DMSO, MTZ^+ 3dpi 2µM MHY1485	unpaired student t-test with Welch's correction	n=13, n=12	p=0.0058
Fig 6F	N=2	MTZ^- 0.04% DMSO, MTZ^- 2µM rapamycin, MTZ^+ 0.04% DMSO	Kruskal-Wallis test;	n=8, n=9, n=9	H=20.85 p<0.0001
		MTZ^- 0.04% DMSO, MTZ^+ 0.04% DMSO	Dunn's multiple comparison test	n=8, n=9	p=0.0265
		MTZ^- 0.04% DMSO, MTZ^- 2µM rapamycin	Dunn's multiple comparison test	n=8, n=9	p=0.1043
	N=2	MTZ^+ 0.04% DMSO, MTZ^+ 2µM rapamycin	unpaired student t-test with Welch's correction	n=8, n=9	p=0.0089
Fig 7H	N=2	0.01% DMSO, 1µM PLX3397	Mann-Whitney test	n=12, n=10	p=0.0001
Fig 7I	N=2	0.01% DMSO, 1µM PLX3397	Mann-Whitney test	n=12, n=10	p=0.0040
Fig 7O	N=1	MTZ^- 0.1% DMSO, MTZ^- 50µM Dex, MTZ^+ 0.1% DMSO	Kruskal-Wallis test;	n=7, n=8, n=6	H=12.31 p=0.0004
		MTZ^- 0.1% DMSO, MTZ^- 50µM Dex	Dunn's multiple comparison test	n=7, n=8	p>0.9999
		MTZ^- 0.1% DMSO, MTZ^+ 0.1% DMSO	Dunn's multiple comparison test	n=7, n=6	p=0.0043
	N=1	MTZ^+ 0.1% DMSO, MTZ^+50µM Dex	Mann-Whitney test	n=6, n=6	p=0.4848
Fig S2 E	N=1	6hpi, 6h UI	Mann-Whitney test	n=6, n=8	p=0.0007

	N=1	2dpi, 7dpf	Mann-Whitney test	n=6, n=9	p=0.0004
Fig S4K	N=1	0.04% DMSO, 2μM rapamycin, 2μM MHY1485	Kruskal-Wallis test;	n=6, n=7, n=7	H=0.07823 p=0.9675
		0.04% DMSO, 2μM rapamycin	Dunn's multiple comparison test	n=6, n=7	p>0.9999
		0.04% DMSO, 2μM MHY1485	Dunn's multiple comparison test	n=6, n=7	p>0.9999
	N=1	0.018% DMSO, 0.9μM INK128	Mann-Whitney test	n=8, n=5	p=0.5952
Fig S4L	N=1	0.04% DMSO, 2μM rapamycin, 2μM MHY1485	Kruskal-Wallis test;	n=6, n=7, n=7	H=2.207 p=0.3450
		0.04% DMSO, 2μM rapamycin	Dunn's multiple comparison test	n=6, n=7	p=0.6486
		0.04% DMSO, 2μM MHY1485	Dunn's multiple comparison test	n=6, n=7	p>0.9999
Fig S5A	N=3	MTZ^- 0.04% DMSO, MTZ^- 2μM rapamycin	Mann-Whitney test	n=7, n=7	0.0041
	N=2	MTZ^- 0.018% DMSO, MTZ^- 0.9μM INK128	Mann-Whitney test	n=6, n=7	0.0012
	N=3	MTZ^+ 0.04% DMSO, MTZ^+ 2μM rapamycin	unpaired student t-test with Welch's correction	n=12, n=15	0.0448
	N=2	MTZ^+ 0.018% DMSO, MTZ^+ 0.9μM INK128	unpaired student t-test with Welch's correction	n=8, n=10	0.0027
Fig S5F	N=1	0.04% DMSO, 2μM rapamycin	unpaired student t-test with Welch's correction	n=8, n=8	0.1746
	N=1	0.018% DMSO, 0.9μM INK128	unpaired student t-test with Welch's correction	n=10, n=9	0.2152
Fig S6	N=3	MTZ^- 0.04% DMSO, MTZ^- 2μM rapamycin	Mann-Whitney test	n=7, n=7	0.0023
	N=2	MTZ^- 0.018% DMSO, MTZ^- 0.9μM INK128	Mann-Whitney test	n=6, n=7	0.0198

	N=3	MTZ^+ 0.04% DMSO, MTZ^+ 2μM rapamycin	unpaired student t-test with Welch's correction	n=12, n=15	0.0179
	N=2	MTZ^+ 0.018% DMSO, MTZ^+ 0.9μM INK128	unpaired student t-test with Welch's correction	n=8, n=10	0.0005
