# Supplementary material for: mTOR activity is essential for retinal pigment epithelium regeneration in zebrafish
Source: PLoS Genet. 2022 Mar 10;18(3):e1009628. doi: 10.1371/journal.pgen.1009628 (PMC8939802; doi:10.1371/journal.pgen.1009628)
Supplement: S10 Table — (PDF) [file pgen.1009628.s018.pdf]

S10 Table. Primers used in this study

Gene	Forward Primer	Reverse Primer
mtor (genotyping)	5′-GATAACGTAGAATGCAGTGGGACAG-3′	5′-CGGGCCCAAACGTATTATGCATAC-3′
scpp8(qRT-PCR)	5′-CGTCTGTCATTTCAAAACCATTGC-3′	5′-AGAGCTCTCATCTGACTCGG-3′
lepb(qRT-PCR)	5′-GGCTCCCGAAGACAGGATAC-3′	5′-AGACATCTGGAAGTGCTCATCT-3′
cxcl18a.1(qRT-PCR)	5′-GCAACAAGGTTCAAATCAAACTCC-3′	5′-AGACCTTCTTCCTCTGTGGGT-3′
ccn1l1(qRT-PCR)	5′-AGAGGCTTATGCAGAGCGGA-3′	5′-TCGCAGGAAGGCTGAAAGTC-3′
