# Supplementary material for: mTOR activity is essential for retinal pigment epithelium regeneration in zebrafish
Source: PLoS Genet. 2022 Mar 10;18(3):e1009628. doi: 10.1371/journal.pgen.1009628 (PMC8939802; doi:10.1371/journal.pgen.1009628)
Supplement: Attachment — Submitted filename: PLOS Genetics rebuttal.pdf [file pgen.1009628.s019.pdf]

This manuscript was reviewed by two experts in the field. Both reviewers found several reasons for the publication of this manuscript, including the importance of the zebrafish model, the novelty of ablating RPE cells using the NTR approach, and the identification of a role for mTOR in RPE regeneration. However, both reviewers also identified a number of major concerns with the data presented and conclusions generated from the data. While there is support for publishing this manuscript in PLOS Genetics, it will require a major revision to deal with the numerous major concerns raised by both reviewers. If the authors are interested in addressing these concerns through additional experiments, the journal would welcome the opportunity to rereview the manuscript.

We are grateful to the Reviewers for their overall enthusiasm with the manuscript and the study, as well as their constructive comments towards improving the work. We have carefully considered each of these here and made major changes to the manuscript to address them. Below, we provide a point-by-point response that outlines these changes, which include additions and clarifications to the text as well as the inclusion of new experiments and data analyses.

For ease, we also highlight new text in the revised manuscript in RED, so the Reviewers can see where these additions have been made.

Reviewer's Responses to Questions

Comments to the Authors:

Please note here if the review is uploaded as an attachment.

Reviewer #1: This paper uses a unique and elegant zebrafish model, where RPE cells are killed by using an NTR approach, to identify a role for mTOR in RPE regeneration. The knowledge they gain understanding the endogenous RPE regenerative response could be helpful in the long-term for the treatment of AMD. Here they extend previous in vitro findings showing that mTOR is required for the survival, migration and proliferation of RPE cells after damage, by moving to an in vivo model. mTOR activity is known to be associated with regeneration in several organs and tissues. The authors show that mTOR is activity in injured RPE, that mTOR inhibition or loss blocks RPE regeneration, and mTOR GOF stimulates the regenerative response. The paper was well written and the data generally nicely presented. The use of both pharmacological inhibitors and an mTOR mutant, the specificity of the NTR model for RPE ablation and its combination with signaling pathway manipulation (LOF and GOF), and the use of transgenic cell specific fluorescent lines are all strengths. These types of approaches are necessary to understand the signaling pathways that control RPE regeneration. Novelty is really restricted to mTOR and the regenerating RPE in vivo, because mTOR is a well-known regulator of regeneration is several organs/tissues, and in vitro data have already implicated mTOR in damaged RPE. While the connection between microglia/macrophages and the mTOR pathway in the RPE after injury is interesting, the data isn't really there to be able to make strong conclusions about the underlying biology.

Major Points:

1) The authors recently published (Leach et al., 2021) that microglia/macrophages respond to RPE injury, and that they are required for the RPE regenerative response. Here they bring in the mTOR pathway, but the advancement is somewhat limited. They aren't able to show that it is mTOR signaling in RPE cells that is required for immune cell recruitment, nor that immune cells regulate mTOR activity in RPE cells. Thus, the conceptual advance in terms of immune cell involvement is somewhat limited.

Specifically, there is some question as to the relationship between mTOR signaling, the RPE, and microglia/macrophage, which reduces the strength of their conclusions.

A) The impairment of recruitment of macrophages/microglia to the injured RPE; is that because of a defect in mTOR signaling in the RPE, or could it be that inhibited mTOR signaling in the immune cells themselves affects their recruitment. The data presented here can't distinguish between the two.

We agree with the Reviewer on this point and have expanded the text discussing this caveat. While our results provide the first in-depth characterization of any molecular underpinning of RPE regeneration, we can not yet differentiate between whether mTOR is required within RPE cells, within leukocytes, or both. Our data do show a requirement for immune cells in reinforcing mTOR activity as PLX3397-mediated depletion of leukocytes prevents the maintenance of

mTOR activity at later stages of the injury response (Fig. 7). Testing this model further requires tissue specific knockout or knockdown approaches (e.g. in either RPE-only or macrophages/microglia-only), which are still fairly difficult to achieve in zebrafish. While these are studies that we hope to perform in the future as we develop the reagents/tools to do so, we do feel that they are beyond the scope of this manuscript to be required here for publication in light of the other conceptual advances reported. Indeed, we are confident that the manuscript, in its revised form, makes a substantial conceptual advance to the field in showing an *in vivo* function for mTOR during RPE regeneration. Very little is known about the RPE regenerative process and outside of zebrafish, there are no animal models in which the process can be studied. Thus, this work also nicely complements our previous work showing cellular requirements for immune cells in modulating the regenerative response [1]. This is where we feel that the mTOR/immune link is an interesting one to note here with the data that we have, and one that should be further pursued in future studies.

B) The analysis in Figure 7 is confusing, and doesn't really argue for macrophage/microglial regulation of mTOR signaling in RPE cells. In comparing panel B and C, there is no apparent eGFP signal in panel C; does that not mean there are no RPE cells? So looking at p-S6 label in C and comparing it to B seems inappropriate. If you have fewer RPE (eGFP+) cells, then one would expect less p-S6. These data seem to argue that in the absence of microglia/macrophages there is greater die-back of the RPE (or delayed regeneration?), but I don't think it says much about p-S6 expression by the RPE, and its regulation by microglia/macrophages. Note that the pink lines are quite dim and hard to see.

We apologize here to the Reviewers; the GFP channel was omitted from Figure 7C when generating the composite Figure. This should have been caught by us when editing and submitting the manuscript, but it was not. Here, we have uploaded a revised figure with the GFP channel included and note that these are the same images as before, just with all channels included. We are very sorry for this omission and the time wasted by the Reviewer due to our mistake.

Pink lines have been changed to white to make them more visible.

2) Not clear to me why in the RNAseq analysis, where RPE cells were FACS-sorted, that there are GABA and neurotransmitter genes that are downregulated. Does this suggest significant contamination of samples with non-RPE neural retina? There should be some assessment of their RNAseq data to indicate how pure it is for RPE cells.

We have added analyses (new S6 Fig) showing expression levels (log2 (CPM+1)) of representative RPE, leukocyte, and retinal cell (including rod and cone photoreceptors, bipolar cells, retinal ganglion cells, amacrine cells, and Muller glia) marker genes across the different treatment conditions examined by RNA-seq. The RPE markers show high expression across all 8 treatment groups relative to other cell type gene markers. By contrast, the expression values of leukocyte and retinal cell marker genes appear relatively low, with the exception of some photoreceptor genes (rho, crx, arr3a, arr3b, opn1sw1, opn1sw2, opn1mw1, opn1mw2). It is well known that the apical microvilli of the RPE interdigitate with photoreceptor outer segments (POS) and that the RPE routinely phagocytose daily-shed POSs. Thus, it is not unreasonable that sorted eGFP+ RPE cells show expression of PR genes due to the close proximity and functionally dependent relationship between the RPE and PRs. This could also be (likely unavoidable) contamination. Regardless, we show robust enrichment of RPE markers in this bulk RNA-seq dataset. With this said, we have added additional text indicating that differentially expressed genes will need additional validation in future studies.

With regard to GABA, this is a well-known neurotransmitter among retinal neurons, but it also acts on RPE cells [2–5]. GABA receptors/transporters were found to be expressed *in vivo* in bullfrog [2], mouse [5], chick RPE [3] and in cultured human RPE cells [4,6], where signaling regulates the intracellular calcium concentration. Calcium signaling is a crucial regulator of tissue regeneration [7] and wound healing [8]. Therefore, it is possible that mTOR-dependent activation of GABA signaling in RPE cells could serve as a trigger to induce migration of regenerating RPE cells and/or reformation of the RPE monolayer. We have added additional discussion on the role of GABA in RPE cells during regeneration to highlight the possibility that these genes are directly functioning during regeneration, but that this awaits experimental validation. This is an exciting area for further study and one that we hope to pursue, but beyond the scope of this study.

3) The rationale for mTOR inhibitor pre-treatment was not provided, and the pre-treatment seems as though it could complicate the authors ability to say that the effects are due exclusively to manipulating mTOR activity after injury. Because of mTOR inhibitor pre-treatment, I would like to see that the extent of the initial injury in the DMSO and mTOR inhibitor treated groups was comparable. The TUNEL data in S3 doesn't focus on RPE TUNEL+ cells, in that the methods suggest these data reflect both cells of the outer nuclear layer and the RPE. Thus, it is hard to know if the RPE injuries are comparable, and at 1 dpi the RPE injury is not yet morphologically evident. If the initial injury is larger in the mTOR inhibitor treated group, looking at the regeneration response with two different injuries is not appropriate. Possibly the RPE regenerative response occurs, but is simply delayed with mTOR inhibition, and this could reflect an initial injury difference.

We have added the rationale for pre-treatment with pharmacological compounds to the Materials and Methods section. Briefly, rapamycin, INK128, or DMSO were added 24 hours prior to ablation to allow these compounds to infiltrate the tissue and take effect before the RPE ablation given the rapid elevation of mTOR activity in response to RPE damage (see Fig. 1).

With respect to the extent of injury in the DMSO and mTOR inhibitor treated groups, we quantified TUNEL staining solely in the RPE at 1dpi (S3 Fig). These data showed no significant differences between $TUNEL^+$ puncta in the RPE of DMSO- and drug-treated groups (0.04% DMSO (n=6) vs rapamycin (n=7; p=0.6486) and vs MHY1485 (n=7; p>0.9999); 0.018% DMSO (n=8) vs INK128 (n=5), p=0.1810). These new statistical comparisons, and more, can be found in Table S9. Moreover, at 1dpi, significant disruption of eGFP+ cell morphology is evident, with eGFP in the RPE appearing discontinuous and accumulating in blebs in both DMSO-treated controls and drug-treated groups. The ONL is also disorganized at this time. These data are consistent with previous observations of RPE and ONL morphology post-ablation [9].

4) Related to the pre-treatment issue raised in point #3, why do the mTOR inhibitor treated eyes (Figures 2C,E,I,K) appear to be much smaller than the DMSO treated counterparts? This does not appear to be the case with the mTOR-/- fish. Does mTOR inhibitor exposure from 4-5 dpf affect eye growth, have non-specific effects, and what consequence could non-RPE related effects of mTOR inhibition have?

To address this question, we measured retinal size of larvae treated with DMSO or mTOR inhibitors from 4-5dpf and from 4dpf - 4dpi. These new data are included in S4 Fig. Quantification revealed no significant decrease in retinal size from short-term (4-5dpf) treatment of larvae, but a significant decrease in overall retinal size in larvae from inhibitor-treated groups when compared to the corresponding DMSO-treated controls. This effect was seen regardless of MTZ treatment. These data indicate that short-term mTOR inhibition doesn't affect eye growth while long-term mTOR inhibition affects eye growth, and that this effect is unrelated to MTZ-induced RPE injury.

mTOR plays a number of well-known roles in modulating cell size and growth of various tissues and organs [10], including in the *Xenopus* CMZ [11]. Decreased eye growth after long-term mTOR inhibition might be related to altered cell behavior of retinal stem cells (RSCs) and retinal progenitor cells (RPCs) in the CMZ. mTOR is an essential regulator of stem and progenitor cell behavior but mTOR function within these populations can be different. For example, in spermatogonial stem cells, it was found that TORC1 activation promotes stem cell differentiation at the expense of self-renewal [12]. In the *Xenopus* retina, mTOR inhibition prevents CMZ-derived progenitor cell differentiation, mimicking nutrient deprivation conditions [11]. In the zebrafish retina/CMZ, it is possible that long-term mTOR inhibition also leads to attenuation of CMZ-derived RPC differentiation to retinal neurons, while increasing RSCs for self-renewal. Indeed, in support of this notion, mTOR inhibition results in increased proliferation within the CMZ (see R1 Minor Comment 3 below and S5 Fig). A similar mechanism has also been recently identified in hematopoietic stem cells where mTOR inhibition results in a loss of quiescence of hematopoietic stem cells and increased proliferation [13]. We have added additional text to this point in the manuscript; however, as our focus here is on RPE regeneration, we do not pursue these CMZ effects further in this study, but it is one of interest for us in future studies focused on CMZ maintenance.

Minor comments:

1) In Figure 1 it is unclear why at 3 hpi in the uninjured (MTZ-) larvae that there is considerable p-S6 label. I thought that the injury to the RPE was via MTZ treatment, and so if there is no MTZ there should be no RPE injury and presumably little or no mTOR response? Essentially, why is the 3 hpi and 6-12 hpi labeling different in panels Fig. 1A vs. Fig. 1B-C?

This was something that we also noted, but didn't explore further at the time of original submission, assuming it was some residual developmental expression that declined rapidly after 5dpf. However, here, we looked into this further and discovered that PTU treatment actually triggers low level mTOR activity. Non-PTU treated larvae showed little to no pS6 labeling at 5dpf, while those treated with PTU showed low staining levels (e.g. Fig. 1 and S1 Fig.). In our experimental paradigm, PTU was washed out at 5dpf/0dpi and this mTOR activity declined in MTZ- animals over time (e.g. at 6 and 12 hpi time points; Fig. 1). Importantly and directly relevant to our study, RPE ablation triggered a robust and statistically significant elevation of pS6 staining in the RPE at 6hpi through 3dpi, when compared to MTZ- controls, indicating that mTOR pathway activity in the RPE is responsive to injury. This is the focus of our study and well supported by these data in Fig. 1, as well as loss- and gain-of-function studies in subsequent Figures. However, the mTOR activity detected as a result of PTU treatment is confounding for any developmental studies performed by others, should they involve PTU. We have added a few sentences addressing this to the Results as a guide for others.

2) Figure 2A experimental design – Rapamycin misspelt

This has been corrected.

3) The CMZ is highly stimulated after MTZ+ and rapamycin treatment and only minimally so after DMSO treatment. What consequence might this have.

Inhibition of mTOR increased proliferation in the CMZ, regardless of ablation status. As noted above (R1 Comment 4), this could reflect direct roles for mTOR activity in RSCs and RPCs of the CMZ. This is an interesting question, but one that requires targeted studies in the CMZ, which is beyond the scope/focus of this study. With regard to its effect on RPE regeneration, our data do not support a role. RPE regeneration is impaired when mTOR is inhibited, despite this increase in CMZ cells. We note in our previous studies [1,9] that we do not yet know the origin of the regenerated RPE. Proliferating cells are detected in the injury-adjacent peripheral RPE early on, and become more centrally localized as RPE regeneration progresses [9]. Thus, our hypothesis is that these injury-adjacent RPE give rise to new RPE and we are testing this through a variety of lineage tracing studies. Alternative hypotheses as to the source of regenerated RPE are Muller glia or RSCs in the CMZ. Preliminary lineage tracing studies do not support either of these origins, but our n's are far too low to yet make any conclusion. That said, in this study, an increased CMZ in mTOR inhibited larvae correlates with decreased RPE regeneration, further suggesting that the CMZ may not be the source of regenerated RPE, as one might expect that regeneration would be enhanced if there were more cells to create an RPE progenitor pool.

There are obviously many caveats to that statement, and we are working through the source of regenerated cells experimentally, again through targeted lineage tracing, but overall, we don't think there are any consequences here on RPE regeneration. Once we know the origin of the regenerated cells, these become directly testable models, but we are not there yet.

4) BrdU cell counts: BrdU data from uninjured eyes should be included for comparison purposes, especially given the pre-treatment with mTOR inhibitors. Representative images should be provided for the BrdU labeling of injured RPE (Figure 2).

These data/quantifications were included in the previous Figure S1. Per the Reviewer's suggestion, we have now added images and moved these data into a revised Figure 2.

5) Line 182: TUNEL data is in Figure S3 not S2, and the MHY1485 data is Figure S2 not S3.

These figure citations have been corrected.

6) In Figure 3 the embryos is it that PTU-treatment has been stopped, and so pigmentation has returned. Is there no worry that the pigmentation of the RPE could impair visualization (quench fluorescence) of more lightly labeled BrdU+ RPE cells?

In all of our BrdU immunohistochemistry images, the BrdU channel was quite bright and cells easily identified. Thus, we aren't worried that the pigmentation impairs visualization of the signal. Regardless, it would affect ablated and unabated larvae of all treatment groups equally and therefore not affect our comparison of overall quantification results.

7) Figure 3I starts at the origin at 60% recovery. The visually big difference in % recovery is a difference of about 8%. The graph should be redrawn with the origin being at 0%.

This has been revised.

8) The BrdU data for the mTOR GOF is separated into a supplemental figure and Figure 4, with only the 3dpi data in Figure 4 being statistically significant. If the argument is that mTOR GOF pushes the peak of RPE proliferation a day earlier, these data should be dealt with together in a single figure. Additionally, this type of time course analysis was not included for the mTOR LOF.

We have combined the previous Fig.4 and Supplementary Fig. S2 into a single figure (new Fig. 4). With regard to the time course, we did not perform this analysis on mTOR loss of function embryos as our previous studies (e.g. [1,9]) used 4dpi as an end-point readout of RPE regeneration, as this is a time when significant regeneration occurs in wild-type and control animals. Given that our hypothesis was that mTOR inhibited the regenerative response, we didn't analyze earlier time points. For MHY1485 treatments, our hypothesis was the converse, that regeneration may be enhanced, and thus, we needed to look earlier, and this is why we examined 2, 3 and 4 dpi.

9) The MHY1485 seems to have disruptive effects on the integrity of the INL of treated retinas (e.g Figure S2G, Figure 4I). Is this a concern?

We have replaced these images as this was an artifact of HCl permeabilization of tissue for BrdU immunohistochemistry. The protocol is very rough on tissue and in some batches, tissue integrity is compromised so during sectioning, tears result. Importantly, this is evident in the images from the former figure (e.g. former Fig. 4G,M) where MHY1485-treated tissue stained for zpr-2, which does not require HCl permeabilization, was normal. In revised Fig. 4, we have replaced these sections with those that possess better tissue integrity and we apologize for the confusion this may have caused with respect to evaluating MHY1485 effects.

10) The RPE recovery data for MHY1485 was performed at 3 dpi and not 4 dpi as in Figures 2 and 3. Is that because by 4 dpi untreated and treated RPE shows comparable recovery?

We first did the BrdU labeling in a time course experiment and found that at 3dpi, there were significantly increased BrdU+ cells in the RPE layer of the MHY1485-treated group (Fig. 4J,K,T). BrdU+ cells at 2dpi (Fig. 4F,G) and 4dpi (Fig. 4N,O) were comparable between MHY1485- and DMSO-treated groups (Fig 4T). Based on these data, we performed the RPE recovery experiments only at 3dpi. We have added additional text to clarify this point.

11) The statistics in graphs where there are multiple treatment groups, and comparisons made between conditions, these should not all be done as t-tests (Figure 7F, S3F). For instance, in Figure S3F these should not be t-tests, but should be an ANOVA, at least when a single control is used for two treatment groups.

For analysis where multiple comparisons between three groups were made, Kruskal–Wallis one-way ANOVA with Dunn's multiple comparison test was used to determine variance and significant difference (please see Table S9 for updated statistical information).

12) How was the mCherry signal measured in Figure 6

For percent area quantification of the mCherry signal in the RPE layer, Regions Of Interest (ROIs) were drawn to encompass the RPE layer using the polygon selection tool in FIJI, which was determined using the DAPI channel to outline the outer edges of photoreceptor outer segments and the bright field channel to outline the basal RPE pigment edges. Thresholding was then performed using the mCherry channel on 8-bit images. The same thresholding parameters were applied to all images in a dataset (e.g. 50/255). Measurements of mCherry signal percent area were made on the central-most sections of each larva. These details have been added to the revised Materials and Methods section.

13) Line 271: there is no Figure 6G

This has been corrected; it is Figure 6F.

14) Without the data to support, actual molecular examples of cytokines should be removed from the model in Figure 8

We agree with the Reviewer and have removed these from Fig. 8.

15) Dexamethasone information needs to be including in Materials and Methods. What is the positive control to show the DEX treatment worked?

Concentrations of dexamethasone (50μM; Sigma-Aldrich) and PLX3397 (1μM; Fisher Scientific) were based on our previous study using the same injury paradigm [1]. The efficacy of Dex has been validated previously in this injury/regeneration model by showing the upregulated expression of *pxr*, a transcriptional target of Dex, after one-day exposure to Dex [1]. Details on Dex concentration and manufacturer can be found in the Materials and Methods section.

16) The BrdU analyses were with a 24 hour pulse, and so it is possible that non-proliferating cells would be counted in the assay.

Any cell during the BrdU exposure period that was proliferating would be labeled under this paradigm. However, this would be the same between all experimental conditions (ablated vs. unablated; treated vs. non-treated) and we always perform assays on control and treatment groups at the same time, so this shouldn't affect our analyses. These 24 hour exposures also served as the foundation for our initial studies [9], which is why we have continued them here.

17) Were measurements done in a manner where the person was blinded to treatment? This type of approach removes subjective bias from measurements, especially when effects are small (e.g. Figure 3I).

They were not (and we did not indicate this in the Methods, to be clear). These quantifications are time intensive and require substantial experience, which creates difficulties when only three members of the lab have this experience (*the three authors of this manuscript*). Thus, quantification for these RPE studies is something we meet as a group to discuss in bi-weekly meetings where we analyze images together, discuss in detail how the images are being analyzed/quantified, what difficulties we are encountering, how we are maintaining consistency across datasets and treatment conditions, etc. We did repeat the analysis in Fig 3K (former 3I) with a blinded individual and the results did not change: MTZ+ *mtor*+/+ (n=9) compared with MTZ+ *mtor*−/− (n=9), unpaired student t-test with Welch's correction, p=0.0080 (unblinded) and p=0.0083 (blinded).

However, recognizing that this is something we need to transition to a fully unbiased and automated process, we have contracted a PhD data scientist with expertise in spatial imaging analyses who is creating a MATLAB-based automated quantification system for these datasets. Our goal is to have this completed and validated so we can include it as part of a *JOVE* collection article on regeneration to which we have been invited to submit in early 2022. The MATLAB code will be freely available once completed so that others can use it.

18) For the mCherry and p-S6 area measurements more detail needs to be provided in the Material and Methods. How was the ROI chosen? What portion of the eye did it include (RPE only?)?

For percent area quantification of p-S6 and mCherry signals in RPE layer, Region Of Interest (ROI) were drawn to encompass the RPE layer using polygon selection tool of Fiji, which was determined using the DAPI channel to outline the outer edges of photoreceptor outer segments and using the bright field channel to outline the basal RPE pigment edges. Thresholding was performed using only on the p-S6 or mCherry channel on 8-bit images under constant thresholds (40/255 and 50/255, respectively).

19) Because these experiments were done on larvae, the authors need to acknowledge that adult RPE could behave somewhat differently.

We agree with the Reviewer and added text in the Discussion to this point.

20) On what basis were the concentrations chosen for drugs, and have they been used in zebrafish previously?

Concentrations of rapamycin (2 μM in DMSO; Sigma-Aldrich), INK128 (0.9 μM in DMSO; Fisher Scientific) and MHY1485 (2μM in DMSO; Sigma-Aldrich) were determined based on levels in system water that allowed larvae to

develop normally from 4dpf-9dpf and whose efficacy was validated through p-S6 immunohistochemistry. Concentrations of dexamethasone (50µM; Sigma-Aldrich) and PLX3397 (1µM; Fisher Scientific) were based on our previous study using zebrafish larvae [1]. This text is included in the Materials and Methods section.

Reviewer #2: This is an interesting manuscript which has potential to reveal novel insights into the mechanism of RPE regeneration in non-mammalian species.

My comments and concerns.

ABSTRACT

-the statement that mTOR activity is sufficient for RPE regeneration needs editing, the evidence may support ability to enhance the process but does not show it is sufficient.

We have changed the words "necessary and sufficient" to "essential".

INTRODUCTION

-to give a balanced view on the status of RPE cell based treatments, it is appropriate to mention the effectiveness reported in clinical trials with RPE patches.

We added text to illustrate the current status of clinical trials with RPE transplantation: "In recent years, RPE cell replacement therapies like transplantation of embryonic or induced pluripotent stem cell-derived RPE, autologous peripheral RPE, or RPE progenitor cells have been shown to restore some visual function in animal models of human disease, and some are in early-phase human trials [14]. For example, clinical trials with RPE patches derived from several different cell sources have revealed preliminary success in safety and efficacy, long-time survival, and improvement in best-corrected visual acuity in AMD patients [15–19]."

RESULTS

-Is p-S6 a specific and selective readout of mTORC1 activation in zebrafish? Is this marker upregulated by other pathways and how do the authors rule these out as driving the p-S6 expression in their model? What is the evidence that the antibody used specifically binds/stains only p-S6 in zebrafish eyes? Has the p-S6 readout been validated by another marker of mTORC1 activation?

p-S6 is a well-established downstream effector of mTORC1 signaling and, yes, it has been used as a readout for mTORC1 activity in zebrafish [20–22], as well as in mice [23,24], chick [25], human tissue [26,27], and in cultured human retinal ganglion cells [28].

S6 is phosphorylated by p70 ribosomal S6 kinase (S6K1 and S6K2) at all serine residues (Ser235, Ser236, Ser240, Ser244, and Ser247) that are subjected to regulation by the mTORC1 signaling pathway. However, several studies described additional protein kinases targeting S6 Ser235 and Ser236 residue independent of of mTORC1/S6K signaling, including p90 ribosomal S6 kinase (RSK), via the extracellular signal-regulated kinase (ERK) signaling [29], protein kinase (PKA) [30], protein kinase B (PKB/AKT) [31], and protein kinase C (PKC)[32], as well as casein kinase 1 targeting S6 residue Ser247 [33]. The p-S6 antibody we used in our study (phospho-S6 Ribosomal Protein (Ser235/236) Rabbit mAb, Cell Signaling #4857) specifically targets ribosomal protein S6 only when phosphorylated at serines 235 and 236, thus we can likely exclude these other pathways that drive p-S6 at other sites. Rapamycin is a specific inhibitor of the mTOR pathway. It specifically blocks phosphorylation and activation by the p70 S6 protein

kinase [34]. In validating this antibody in our study, we demonstrate that p-S6 in the 2dpi RPE was completely abolished by rapamycin, as well as by INK128 treatment (Fig 2A-E), supporting the notion that the p-S6 activity in Fig 1 results from activated mTOR signaling. We also performed negative staining controls, which we stained with p-S6 primary antibody and no secondary antibody, or secondary antibody only, and no signal was detected in the RPE.

-What age and at what time of day does the MTZ induced ablation start? Is this always consistent?

All ablations in this study were performed at 5dpf, for exactly 24 hours (i.e. larvae are always collected at the precise time from injury indicated). This is described in the Material and Methods and Results/Figures. Ablations are not always performed at the same time of day on different days of experimentation (i.e. on an exact circadian cycle). However, these are generally initiated within a window of a few hours of each other across different experimentation days based on when the animals breed, and always for 24 hours.

-If MTZ- larvae are unablated, it is confusing in parts that the analysis is referred to as hpi (if not injured)

We apologize for this confusion; these time points are matched to those in the ablated larvae, but we are unsure what the clearest way to label these is. For example, 3hpi is 5.125 dpf, 6hpi is 5.25 dpf, and so on. This seemed cumbersome to use, so we tried to use MTZ- and MTZ+ as the descriptors for uninjured and injured. In this revision, we've tried a new way, where we label them as 3hpi UI, with UI defined in the text as uninjured. We don't know if this makes it clearer or more confusing and are open to any suggestions from a reviewer (reader) as to how best to display these times.

-There are high levels of pS6+ in the uninjured eyes/RPE that steadily decreases out to 4 days pi (Fig 1 O). Why do such changes occur if there is no intervention/injury? Is p-S6 dynamically expressed in the eye during the eye or during development from 5-9 dpf?

Please see our response to R1, minor comment 1, as it also relates to p-S6 levels at 3hpi in the uninjured (UI) eye and PTU treatment/removal. Importantly, the relevant part of our analyses here is that p-S6 levels increase significantly in the MTZ+ eye, relative to those in the uninjured eye, from 6hpi to 3dpi. This, and subsequent studies, show the importance of mTOR signaling during RPE regeneration. Whether mTOR is active dynamically during development, or larval growth, is an interesting question but not one that we pursue in this manuscript.

-The levels of marker staining in the RPE is a key measure used to interpret the data. More specific information is needed to know how staining in the RPE is segregated and quantified from other tissues. Furthermore, if there is pS6 staining in macrophages/microglia that have been recruited to the RPE, how is the staining levels attributed to RPE cells?

For quantification of marker expression in the RPE layer, regions of interest (ROIs) were drawn to encompass the RPE layer using the polygon selection tool in FIJI. ROIs were determined using the DAPI channel to outline the outer edges of photoreceptor outer segments and the bright field channel to outline the basal RPE pigment edges. This is clarified in the Materials and Methods section.

p-S6 quantifications are performed relative to the RPE layer, not specifically RPE cells. At 2 and 3dpi, when there was significant infiltration of macrophages/microglia in the RPE layer [1], the p-S6 signaling we quantified reflected mTOR activity in both RPE cells and infiltrated macrophage/microglia in RPE layer. Staining is not attributed to RPE cells only, and the text is careful to this point, always stating relative to the RPE layer, which, as noted above, is segregated in each image prior to quantification.

In many of the drug treated samples, the eye morphology appears to be regressed compared to controls (e.g. Fig 2E, N, P, 4C, P). This suggests there developmental delays or toxicity induced by the drugs which would confound interpretation of the data. What evidence counters this explanation?

We addressed this with Reviewer 1, see major comment 4 above. Briefly, we measured retinal size (yellow line in S3 Fig) of larvae treated with DMSO or mTOR inhibitors from 4-5dpf and from 4dpf - 4dpi. These new data are included in S4 Fig. Quantification revealed no significant decrease in retinal size from short-term (4-5dpf) treatment of larvae, but a significant decrease in overall retinal size in larvae from inhibitor-treated groups when compared to the corresponding DMSO-treated controls. This effect occurred regardless of MTZ treatment. These data indicate that short-term mTOR

inhibition doesn't affect eye growth while long-term mTOR inhibition affects eye growth, and that this effect is unrelated to MTZ-induced RPE injury.

mTOR plays a number of well known roles in modulating cell size and growth of various tissues and organs [10], including in the *Xenopus* CMZ [11]. Decreased eye growth after long-term mTOR inhibition might be related with altered cell behavior of retinal stem cells (RSCs) and retinal progenitor cells (RPCs) in the CMZ. mTOR is an essential regulator of stem and progenitor cell behavior but mTOR function within these populations can be different. For example, in spermatogonial stem cells, it was found that TORC1 activation promotes stem cell differentiation at the expense of self-renewal [12]. In the *Xenopus* retina, mTOR inhibition prevents CMZ-derived progenitor cell differentiation, mimicking nutrient deprivation conditions [11]. In the zebrafish retina/CMZ, it is possible that long-term mTOR inhibition also leads to attenuation of CMZ-derived RPC differentiation to retinal neurons, while increasing RSCs for self-renewal. Indeed, in support of this notion, mTOR inhibition results in increased proliferation within the CMZ (see R1 Minor Comment 3 below and S5 Fig). A similar mechanism has also been recently identified in hematopoietic stem cells where mTOR inhibition results in a loss of quiescence of hematopoietic stem cells and increased proliferation [13]. We have added additional text to this point in the manuscript; however, as our focus here is on RPE regeneration, we do not pursue these CMZ effects further in this study, but it is one of interest for us in future studies focused on CMZ maintenance.

-I'm concerned that the increased levels of marker staining with the mTOR activator (e.g. pS6) at later time points (e.g. 3 dpi) actually reflects a delay/toxic effect to the fish which are now stalled in the 12 hpi stage when the peak occurs in the model. In other words the elevation at 3 dpi is actually due to the fish been delayed and aged around the peak of staining that occurs 12 hpi.

We assume that the Reviewer means 2dpi, which is the time at which we detect significantly elevated p-S6 in MHY1485-treated larvae. Our data do not suggest a delay in development, however. Retina size is not significantly different in MHY1485-treated larvae when compared to DMSO controls (Reviewer Fig. 1). Cell death is also not elevated at 1dpi in MHY1485-treated larvae (S3L Fig.). Together, these support a model in which MHY11485 enhances mTOR activity in the injured RPE and this, in turn, facilitates enhanced regeneration as shown in Fig. 4.

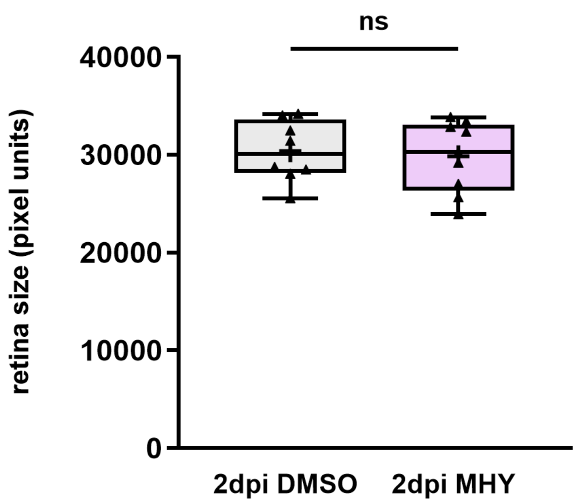

Reviewer Figure 1: Retinal size in 2dpi DMSO (n=8) vs 2dpi MHY1485-treated (n=9) is not significantly different. Unpaired student t-test with Welch's correction, p= 0.7488.

-As RNAseq analysis in zebrafish is notorious for false positives, the authors need to provide data validating the RNAseq by qPCR.

We're aware of no published evidence that RNA-seq in zebrafish is more prone to false positives than in other systems/models. We also respectfully disagree about the need for additional validation of our RNA-seq dataset. This is a hypothesis generating experiment, not an end result, and we carefully tested/validated one hypothesis resulting from our data - that immune responses were modulated by the mTOR pathway. There are many additional hypotheses

that come from this dataset, each of which needs to be carefully validated by experiments specific to the gene or pathway involved. Here, we include three biological replicates for each group and we filtered (log2 fold change>1; FDR p-value<0.05, max group mean≥1) DEGs to exclude the genes with small differences in expression.

The issue of needing additional validation is one that we have thought of quite a lot over the last few years, as most of our studies involved using RNA-seq to generate hypotheses. It is also one that has been addressed in a number of recent editorials as well [35]. It is not clear to us that qPCR validation provides any additional rigor to a study unless there are insufficient replicates or a model is based on differential expression of only a few genes with low expression levels and/or with differences in expression that are quite small. Put differently, we don't see how simply picking a few DEGs and doing qPCR is a robust validation system, nor how it addresses the data set as a whole where hundreds to thousands of transcripts change expression under each condition and at varying statistical thresholds. Philosophically (and probably at the risk of opening a larger debate that is unrelated to the contents of this manuscript, which is not our intent), we question whether small changes that are validated as statistically significant in a dataset are in fact biologically relevant and important, which can really only be determined experimentally with a thorough characterization. One can easily throw in a qPCR graph showing that five "candidate" genes validate in the direction and approximate scale predicted by the RNA-seq data, but in a large data set with many candidates, this cannot engender any more confidence as to the fidelity of the data set as a whole nor does it have much meaning biologically without further experimentation (an issue nicely addressed in [36]). There are obvious caveats to that statement, but our point is that by simply cherry picking a few candidates and showing that they behave in qPCR as predicted by the RNA-seq adds no additional rigor or biological insight to a story. We selected one candidate pathway here and rigorously validated that experimentally as important and this is the type of follow-up study that is necessary for any putative candidate gene/pathway to be "validated".

-What QC was performed to confirm that the FACS cells are RPE purified/enriched? Are all the genes known to be expressed in the RPE? Are there any genes identified that are known not to be expressed in the RPE? Are the invading microglia/macrophages excluded from the purified cells?

We address this to some extent with Reviewer 1, Comment 2. Briefly, we have added analyses (new S6 Fig) showing expression levels (log2 (CPM+1)) of representative RPE, leukocyte, and retinal cell (including rod and cone photoreceptors, bipolar cells, retinal ganglion cells, amacrine cells, and Muller glia) marker genes across the different treatment conditions examined by RNA-seq. RPE markers show high expression across all 8 treatment groups relative to other cell type gene markers, with the exception of some photoreceptor genes (rho, crx, arr3a, arr3b, opn1sw1, opn1sw2, opn1mw1, opn1mw2). This could be due to the phagocytic activity of RPE or contamination. Regardless, we show robust enrichment of RPE markers in this bulk RNA-seq dataset and these data were informative in identifying a link with immune responses, which we pursue and validate in further experiments in the manuscript.

-In Fig 2, why is there a need for Panel B and D (they appear as the same expt). Same for panel H and J.

These reflect different concentrations of DMSO. This has been made clear on the Figure and in the Figure legends.

-The figures need more details as to the number of biological replicates (fish) and number of independent experiments used.

Detailed statistics are listed in Table S9, which includes numbers of independent experiments for each dataset, the statistical test performed for each comparison, the number of biological replicates (larvae), and exact p-values. We added additional text to each figure legend to guide readers to Table S9 for statistical information.

-In order to make conclusions re inflammation-independent regeneration, the authors needed to demonstrate that Dex gets into the zebrafish eye/ has a biological effect in the eye at this timeline.

The efficacy of Dex has been validated previously in this injury/regeneration model by showing the upregulated expression of *pxr*, a transcriptional target of Dex, after one-day exposure to Dex [1]. Details on Dex concentration and manufacturer information can be found in the Materials and Methods section.

REBUTTAL REFERENCES

1. Leach LL, Hanovice NJ, George SM, Gabriel AE, Gross JM. The immune response is a critical regulator of

zebrafish retinal pigment epithelium regeneration. Proc Natl Acad Sci U S A. 2021;118. doi:10.1073/pnas.2017198118

2. Peterson WM, Miller SS. Identification and functional characterization of a dual GABA/taurine transporter in the bullfrog retinal pigment epithelium. J Gen Physiol. 1995;106: 1089–1122.

3. Cheng Z-Y, Wang X-P, Schmid KL, Liu L. Identification of GABA receptors in chick retinal pigment epithelium. Neurosci Lett. 2013;539: 43–47.

4. Cheng Z-Y, Wang X-P, Schmid KL, Han X-G, Song H, Tang X. GABAAα1 and GABAAρ1 subunits are expressed in cultured human RPE cells and GABAA receptor agents modify the intracellular calcium concentration. Mol Vis. 2015;21: 939–947.

5. Blanks JC, Roffler-Tarlov S. Differential localization of radioactive gamma-aminobutyric acid and muscimol in isolated and in vivo mouse retina. Exp Eye Res. 1982;35: 573–584.

6. Booij JC, ten Brink JB, Swagemakers SMA, Verkerk AJMH, Essing AHW, van der Spek PJ, et al. A new strategy to identify and annotate human RPE-specific gene expression. PLoS One. 2010;5: e9341.

7. Liu Y, Lou WP-K, Fei J-F. The engine initiating tissue regeneration: does a common mechanism exist during evolution? Cell Regen. 2021;10: 12.

8. Ghilardi SJ, O'Reilly BM, Sgro AE. Intracellular signaling dynamics and their role in coordinating tissue repair. Wiley Interdiscip Rev Syst Biol Med. 2020;12: e1479.

9. Hanovice NJ, Leach LL, Slater K, Gabriel AE, Romanovicz D, Shao E, et al. Regeneration of the zebrafish retinal pigment epithelium after widespread genetic ablation. PLoS Genet. 2019;15: e1007939.

10. Saxton RA, Sabatini DM. mTOR Signaling in Growth, Metabolism, and Disease. Cell. 2017;168: 960–976.

11. Love NK, Keshavan N, Lewis R, Harris WA, Agathocleous M. A nutrient-sensitive restriction point is active during retinal progenitor cell differentiation. Development. 2014;141: 697–706.

12. Hobbs RM, Seandel M, Falciatori I, Rafii S, Pandolfi PP. Plzf regulates germline progenitor self-renewal by opposing mTORC1. Cell. 2010;142: 468–479.

13. Fan C, Zhao C, Zhang F, Kesarwani M, Tu Z, Cai X, et al. Adaptive responses to mTOR gene targeting in hematopoietic stem cells reveal a proliferative mechanism evasive to mTOR inhibition. Proc Natl Acad Sci U S A. 2021;118. doi:10.1073/pnas.2020102118

14. Chichagova V, Hallam D, Collin J, Zerti D, Dorgau B, Felemban M, et al. Cellular regeneration strategies for macular degeneration: past, present and future. Eye . 2018;32: 946–971.

15. da Cruz L, Fynes K, Georgiadis O, Kerby J, Luo YH, Ahmado A, et al. Phase 1 clinical study of an embryonic stem cell-derived retinal pigment epithelium patch in age-related macular degeneration. Nat Biotechnol. 2018;36: 328–337.

16. Karasu B, Erdoğan G. Autologous translocation of the choroid and retina pigment epitelial cells(RPE) in age-related macular degeneration: Monitoring the viability of choroid and RPE patch with indocyanine green angiography(ICGA) and fundus autofluorescence(FAF). Photodiagnosis Photodyn Ther. 2019;28: 318–323.

17. Schwartz SD, Hubschman J-P, Heilwell G, Franco-Cardenas V, Pan CK, Ostrick RM, et al. Embryonic stem cell trials for macular degeneration: a preliminary report. Lancet. 2012;379: 713–720.

18. Kashani AH, Lebkowski JS, Rahhal FM, Avery RL, Salehi-Had H, Dang W, et al. A bioengineered retinal pigment epithelial monolayer for advanced, dry age-related macular degeneration. Sci Transl Med. 2018;10. doi:10.1126/scitranslmed.aao4097

19. Mandai M, Watanabe A, Kurimoto Y, Hirami Y, Morinaga C, Daimon T, et al. Autologous Induced Stem-Cell-Derived Retinal Cells for Macular Degeneration. N Engl J Med. 2017;376: 1038–1046.

20. Zhang Z, Hou H, Yu S, Zhou C, Zhang X, Li N, et al. Inflammation-induced mammalian target of rapamycin signaling is essential for retina regeneration. Glia. 2020;68: 111–127.

21. Diekmann H, Kalbhen P, Fischer D. Active mechanistic target of rapamycin plays an ancillary rather than essential

role in zebrafish CNS axon regeneration. Front Cell Neurosci. 2015;9. doi:10.3389/fncel.2015.00251

22. He J, Chen J, Wei X, Leng H, Mu H, Cai P, et al. Mammalian Target of Rapamycin Complex 1 Signaling Is Required for the Dedifferentiation From Biliary Cell to Bipotential Progenitor Cell in Zebrafish Liver Regeneration. Hepatology. 2019;70: 2092–2106.

23. Pita-Thomas W, Mahar M, Joshi A, Gan D, Cavalli V. HDAC5 promotes optic nerve regeneration by activating the mTOR pathway. Exp Neurol. 2019;317: 271–283.

24. Volovelsky O, Cohen G, Kenig A, Wasserman G, Dreazen A, Meyuhas O, et al. Phosphorylation of Ribosomal Protein S6 Mediates Mammalian Target of Rapamycin Complex 1-Induced Parathyroid Cell Proliferation in Secondary Hyperparathyroidism. J Am Soc Nephrol. 2016;27: 1091–1101.

25. Zelinka CP, Volkov L, Goodman ZA, Todd L, Palazzo I, Bishop WA, et al. mTor signaling is required for the formation of proliferating Müller glia-derived progenitor cells in the chick retina. Development. 2016;143: 1859–1873.

26. Arenas DJ, Floess K, Kobrin D, Pai R-AL, Srkalovic MB, Tamakloe M-A, et al. Increased mTOR activation in idiopathic multicentric Castleman disease. Blood. 2020;135: 1673–1684.

27. Liu J, Reeves C, Michalak Z, Coppola A, Diehl B, Sisodiya SM, et al. Evidence for mTOR pathway activation in a spectrum of epilepsy-associated pathologies. Acta Neuropathol Commun. 2014;2: 71.

28. Teotia P, Van Hook MJ, Fischer D, Ahmad I. Human retinal ganglion cell axon regeneration by recapitulating developmental mechanisms: effects of recruitment of the mTOR pathway. Development. 2019;146. doi:10.1242/dev.178012

29. Roux PP, Shahbazian D, Vu H, Holz MK, Cohen MS, Taunton J, et al. RAS/ERK signaling promotes site-specific ribosomal protein S6 phosphorylation via RSK and stimulates cap-dependent translation. J Biol Chem. 2007;282: 14056–14064.

30. Biever A, Puighermanal E, Nishi A, David A, Panciatici C, Longueville S, et al. PKA-dependent phosphorylation of ribosomal protein S6 does not correlate with translation efficiency in striatonigral and striatopallidal medium-sized spiny neurons. J Neurosci. 2015;35: 4113–4130.

31. Yano T, Ferlito M, Aponte A, Kuno A, Miura T, Murphy E, et al. Pivotal role of mTORC2 and involvement of ribosomal protein S6 in cardioprotective signaling. Circ Res. 2014;114: 1268–1280.

32. House C, Wettenhall RE, Kemp BE. The influence of basic residues on the substrate specificity of protein kinase C. J Biol Chem. 1987;262: 772–777.

33. Hutchinson JA, Shanware NP, Chang H, Tibbetts RS. Regulation of ribosomal protein S6 phosphorylation by casein kinase 1 and protein phosphatase 1. J Biol Chem. 2011;286: 8688–8696.

34. Chung J, Kuo CJ, Crabtree GR, Blenis J. Rapamycin-FKBP specifically blocks growth-dependent activation of and signaling by the 70 kd S6 protein kinases. Cell. 1992;69: 1227–1236.

35. Coenye T. Do results obtained with RNA-sequencing require independent verification? Biofilms. 2021;3: 100043.

36. Hughes TR. “Validation” in genome-scale research. J Biol. 2009;8: 3.
