# Supplementary material for: mTOR activity is essential for retinal pigment epithelium regeneration in zebrafish
Source: PLoS Genet. 2022 Mar 10;18(3):e1009628. doi: 10.1371/journal.pgen.1009628 (PMC8939802; doi:10.1371/journal.pgen.1009628)
Supplement: Attachment — Submitted filename: PLOS Genetics rebuttal.pdf [file pgen.1009628.s020.pdf]

While the revised manuscript has been improved and the authors have addressed several of the previous comments, Reviewer 2 makes several important and compelling points that need to be addressed by the reviewers in this manuscript. Please carefully consider the comments of Reviewer 2 and address each experimentally or with a thoughtful rebuttal.

This manuscript was re-reviewed by two experts in the field. Reviewer 1 had no additional comments, while Reviewer 2 requested a few additional changes. We have carefully considered each of these here and made changes to the manuscript to address them. Below, we provide a point-by-point response that outlines these changes, which include additions and clarifications to the text as well as the inclusion of new experiments and data analyses.

For ease, we also highlight new text in the revised manuscript in RED, so the Reviewers can see where these additions have been made.

<u>Reviewer #1:</u> My comments to authors are restricted to the following. The authors have reasonably addressed my concerns.

<u>Reviewer #2:</u> This is an interesting manuscript which has potential to reveal novel insights into the mechanism of RPE regeneration in non-mammalian species.

My updated comments and concerns.

RESULTS

*****I don't comprehend how you can "likely exclude" these other pathways if they can also phosphorylate the same Ser residues. Has the p-S6 readout been validated by another marker of mTORC1 activation?**

I think our response was perhaps not worded as precisely as it should have been and for that we apologize.

Our logic that p-S6 is specific is as follows:

p-S6 is a well-established downstream effector of mTORC1 signaling that has been used as a readout for mTORC1 activity in zebrafish [1–3], as well as in mice [4,5], chick [6], human tissue [7,8], and in cultured human retinal ganglion cells [9]. S6 is phosphorylated by p70 ribosomal S6 kinase (S6K1 and S6K2) at all serine residues (Ser235, Ser236, Ser240, Ser244, and Ser247), and these kinases are regulated by the mTORC1 signaling pathway. However, several studies described additional protein kinases capable of targeting S6 Ser235 and Ser236 residues independent of mTORC1/S6K signaling. These include: p90 ribosomal S6 kinase (RSK), via the extracellular signal-regulated kinase (ERK) signaling pathway [10]; protein kinase (PKA) [11]; protein kinase B (PKB/AKT) [12]; and protein kinase C (PKC)[13]. The p-S6 antibody we used in our study (phospho-S6 Ribosomal Protein (Ser235/236) Rabbit mAb, Cell Signaling #4857) specifically targets ribosomal protein S6 only when phosphorylated at serines 235 and 236.

Here, we demonstrate that p-S6 in the 2dpi RPE was completely abolished by rapamycin and INK128 treatments (Fig 2A-E), as well as in an *mtor* mutant (Fig 3A-C), supporting the notion that the p-S6 activity in Fig 1 results from activated mTOR signaling. We showed the opposite effect, that p-S6 is increased at 2dpi, in the presence of MHY1485, a known mTOR activator (Fig 4B,C,R). We also performed negative staining controls, which we stained with p-S6 primary antibody and no secondary antibody, or secondary antibody only, and no signal was detected in the RPE. We do not have an additional readout of mTORC1 activation at this time that works in zebrafish, but given the respective decrease or increase in p-S6 staining with mTOR-specific inhibitors/mutants or MHY1485 treatment and careful negative control experiments done here, coupled with what is already known about rapamycin as a specific inhibitor of the mTOR pathway that blocks phosphorylation and activation of S6 by the S6K, but not other kinases [14], it is unlikely that these other pathways contribute to the p-S6 staining detected after RPE ablation.

*****In the methods, state the time window used.**

Text has been added to the methods to clarify the time window used. At 5dpf, larvae were treated with 10mM metronidazole (MTZ) in system water between 10am-2pm. 24 hours later, larvae were removed from MTZ and maintained in system water until euthanized for experiments.

*****I agree this is difficult, and that the new labelling is still confusing. A schematic in Fig 1 would clarify the design and the label meaning.**

We added the following schematic to a revised Figure 1 to clarify time points/nomenclature.

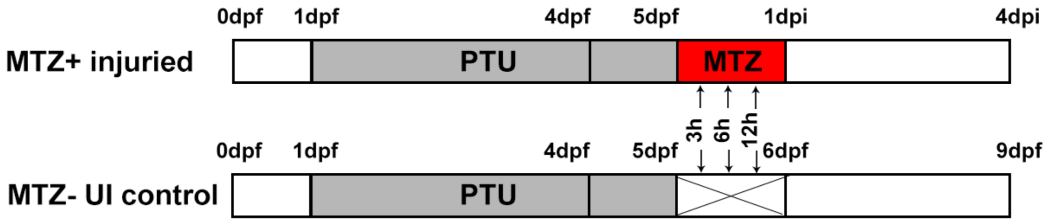

*****Can the authors show that in WT fish, MTZ does not induce higher pS6+?**

To address this issue, we performed a new experiment, treating WT fish with MTZ at 5dpf and then assaying p-S6 levels 6 hours later and 2 days later, times at which p-S6 is significantly elevated in MTZ-treated *rpe65a*:nfsB-eGFP transgenic fish. MTZ did not elicit p-S6 signal in the RPE of MTZ-treated WT larvae at either time point. These new data have been added as S2 Figure in the revised manuscript.

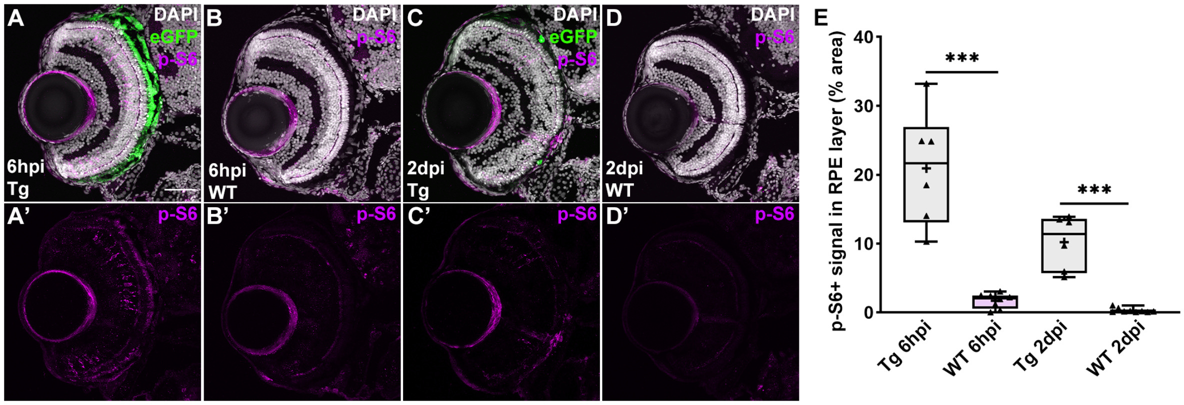

S2 Fig. MTZ treatment does not elicit mTOR activity in the RPE layer of wild-type larvae.

(A-D) Immunofluorescent images of p-S6 staining on transverse cryosections from (A,C) MTZ-treated larvae carrying rpe65a:nfsB-eGFP transgene (MTZ+ Tg) and (B,D) MTZ-treated wild-type larvae (MTZ+ WT). Nuclei (white), p-S6 (magenta), eGFP (green). (A'-D') Single channel immunofluorescent images of p-S6. (E) p-S6 signals in the RPE layer were significantly elevated post-MTZ treatment (6hpi and 2dpi) in rpe65a:nfsB-eGFP Tg larvae when compared to WT controls. Statistical information can be found in S9 Table. Dorsal is up and distal is left. Scale bar = 50μm.

*****While I appreciate the comprehensive response, I don't consider that this definitively locks affects of the drugs to changes in RPE regeneration. The assay endpoint is as 9 dpf, so even if the drugs have no obvious effect on eye development from 4-5 dpf, they do from 4-9 dpf. If there is a "general ocular toxicity" with the drugs that may explain the reduced BrdU levels in the RPE at 9 dpf, and not a specific effect on mTOR in RPE regeneration. Have the authors shown that if they add the mTOR drugs late (e.g. 1-2 dpi) that they still reduce BrdU levels in the "regenerating RPE"?**

To address this concern (coupled with the information provided in our previous response regarding the increased proliferation we detect in the CMZ of rapamycin-treated larvae), we performed the experiment suggested by the Reviewer and treated regenerating larvae with rapamycin from either 1-4dpi or 2-4dpi. BrdU levels were significantly reduced after both treatments. These data suggest that sustained mTOR activity is required for RPE regeneration. Indeed, p-S6 is significantly elevated after RPE ablation through 3dpi. This is an interesting finding and one that requires additional follow up that is beyond the scope of this manuscript. We include the data here as a Reviewer Figure.

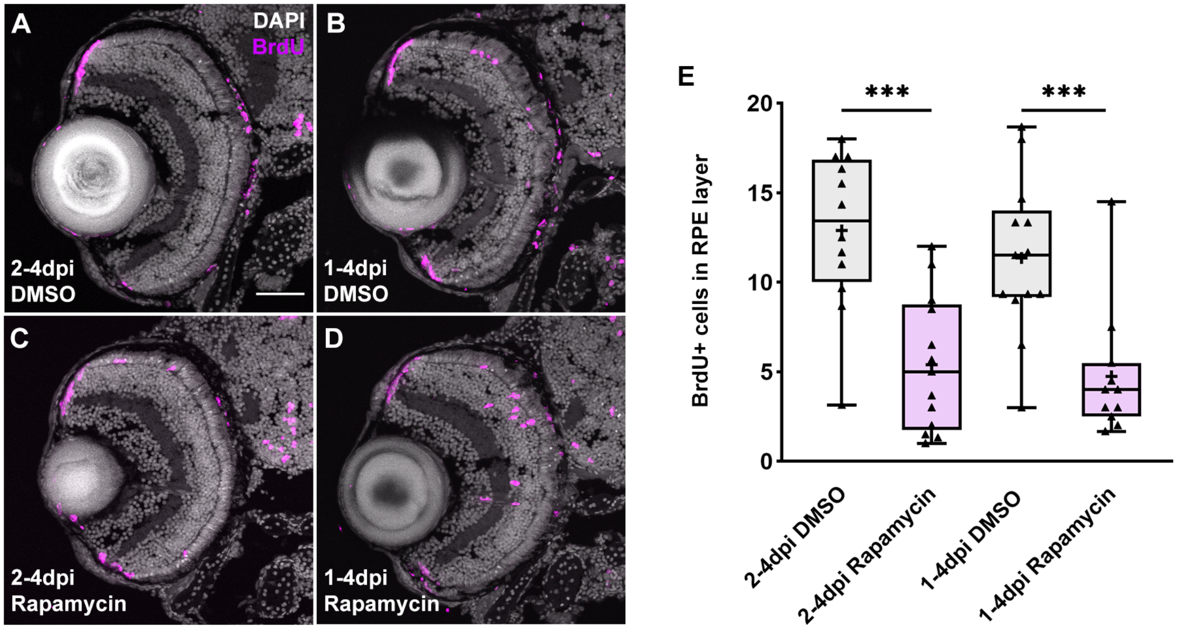

Reviewer Figure 1: Rapamycin inhibition of mTOR activity from 1-4dpi and 2-4dpi impairs proliferation in the RPE layer.

(A-D) Immunofluorescent images of BrdU staining on transverse cryosections from ablated DMSO- and rapamycin--treated larvae (A,C) from 1dpi to 4dpi and (B,D) from 2dpi to 4dpi. Nuclei (white), BrdU (magenta), eGFP (green). (E) Quantification of BrdU+ cells in the RPE layer revealed significant decreases in both 1-4pi (DMSO, n=13, rapamycin, n=11; Mann-Whitney test, p=0.0008); and 2-4dpi (DMSO, n=13; rapamycin, n=13, unpaired student t-test with Welch's correction, p=0.0001) time windows after treatment with rapamycin, when compared to corresponding DMSO-treated controls. Dorsal is up and distal is left. Scale bar = 50µm.

*****I agree that qPCR validation of a small number of genes may have little meaning biologically. However, I am aware from others that there are issues with mis-mapping of reads in zebrafish RNAseq data sets. In order, to provide technical validation of the dataset so that the community can be confident in using it as a point to test hypotheses, I think it is very reasonable to show validation of a small cohort of genes. If they validate, as you expect, that provides confidence to everyone.**

As requested by the Reviewer, we have added qPCR data validating the RNA-Seq data for *scpp8, cxcl18a.1, lepb, ccn1l1* from 2dpi/7dpf RNA sequencing dataset in two biological replicates (new S8 Figure). Reviewer Table 1 shows the raw TPM (transcripts per million) data across four treatment conditions in 3 biological replicates from the RNA sequencing dataset. These four genes were significantly upregulated in the MTZ+ DMSO group, compared to the MTZ- DMSO group, and significantly downregulated in the MTZ+ rapamycin group, compared with the MTZ+ DMSO control group (S1-S4 Table). Here, qPCR quantification of normalized gene expression in cDNA isolated from Replicates 1 and 2 (Replicate 3 was not able to be retrieved for qPCR) of the FACS-isolated RPE cell samples used for RNA-Sequencing demonstrates that changes in gene expression of all four genes matches that determined from RNA sequencing. Therefore, q-PCR results are in accordance with gene expression changes identified through RNA-sequencing, providing technical validation. Primer sequences utilized have been added in a new S10 Table.

Finally, we are also compelled to note that it is completely inappropriate for a Reviewer to use the statement, "*I am aware from others*……." as the basis of a criticism in peer-review. This is not appropriate in a manuscript review, grant review, or any other peer-reviewed forum. We provided access to all of our raw data for review; if there was an issue with our analyses, the Reviewer should note it; if there was an issue with our methodology, the Reviewer should provide direct criticism of the method in question and/or published studies refuting our approach. This Reviewer has done neither of these. *PLoS Genetics* states in their Guidelines for Authors that "*PLOS does not accept references to "data not shown."*" PLOS Reviewers should be held to the same standards.

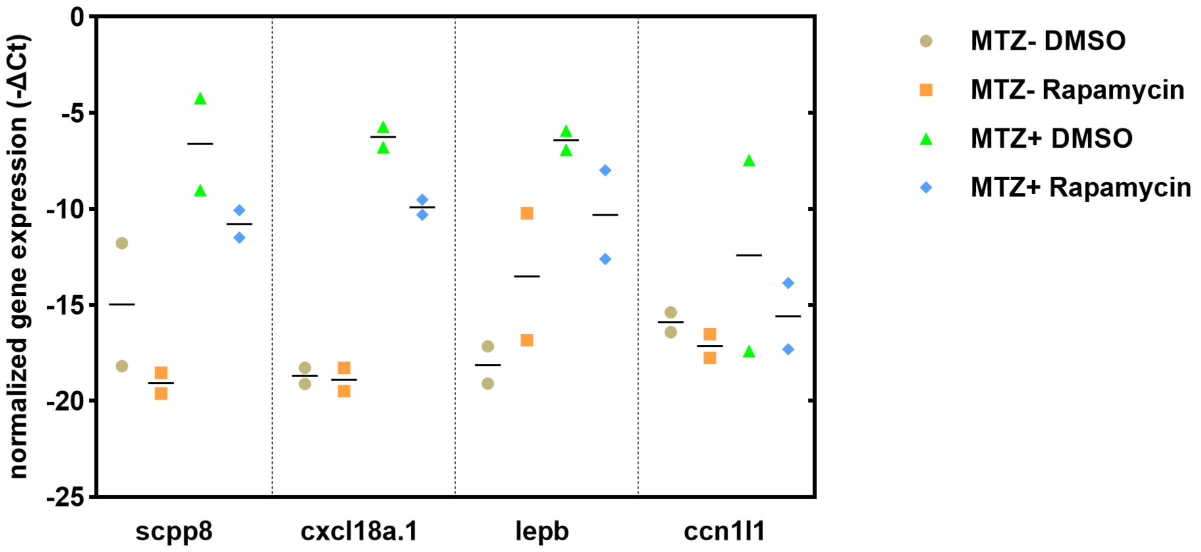

<u>S8 Fig:</u> Quantitative real-time PCR analysis of scpp8, cxcl18a.1, lepb, ccn1l1 gene expression to validate 2dpi/7dpf RNA-sequencing results.

qRT-PCR validation of four differentially expressed genes (scpp8, cxcl18a.1, lepb, ccn1l1) in MTZ- DMSO, MTZ-Rapamycin, MTZ+ DMSO, and MTZ+ Rapamycin treatment groups used for RNA-seq at 2dpi/7dpf. Similar to RNA-seq results (see Fig 5D, S1 Table, S6 Table), data show increased expression of all genes in MTZ+ DMSO groups (green) compared to MTZ- DMSO (tan) and MTZ+ Rapamycin groups (blue). Transcript levels were normalized to the expression of beta-actin. Lines represent the median. Each data point represents a biological replicate (n=2).

Gene	MTZ- DMSO (TPM)			MTZ- Rapamycin (TPM)			MTZ+DMSO (TPM)			MTZ+ Rapamycin (TPM)		
	R1	R2	R3	R1	R2	R3	R1	R2	R3	R1	R2	R3
lepb	0	0	0	2.25	0.09	0	73.39	49.72	103.35	14.92	0.8	0.9
ccn1l1	0	0	0	0	0	0	33.81	0.42	32.78	1.71	0.53	0.17
scpp8	0.5	0	0.5	0	0.03	0.06	150.32	8.38	22.96	1.49	0.44	0.25
cxcl18a.1	0	0	0	0	0	0	33.15	19.67	2.72	1.41	1.18	3.18

TPM: transcripts per million; R: Biological Replicate

Reviewer Table 1. Expression values of *lepb, ccn1l1, scpp8, cxcl18a.1* genes from 2dpi/7dpf RNA-Sequencing dataset

*****The paper cited does show increased pxr after Dex treatment, but this was in larvae, not in eyes, and therefore does not adequately address the original comment.**

The Reviewer is correct in noting that we did not assess Dex efficacy in RPE samples, but have done so systemically using *pxr* as a readout [15]. Given the limited time afforded for this revision and repair and staffing issues with the cell sorter used in this study, we were not able to FACS isolate RPE cells in Dex-treated animals and do qPCR in these samples. With that said, numerous publications have used Dex and validated its efficacy in CNS tissues. These include Silva et al., 2020 [16], which showed that bathing fish in Dex resulted in decreased expression of inflammatory genes in the eye after injury, Caldwell et al. which showed efficacy in the diencephalon [17] and the publication cited by most of these, Kyritsis et al., 2012, which showed effects in the telencephalon [18]. In addition to these, many other studies have shown the effects of Dex on CNS regeneration, through modifications of the Kyritsis et al., 2012 protocol, though without direct validation. These include White et al., 2017 [19], Zhang et al., 2020 [1] and Iribarne and Hyde, 2021 [20], each of which focused on Muller glia and retinal regeneration, showing Dex effects on immune cells or their behavior, but without a direct analysis of Dex efficacy through target gene expression. Taken together, the route of administration and concentrations of Dex used, coupled to previous work by our lab [15] and these other labs showing that Dex exposures are effective in zebrafish, supports our use of Dex and data included in Fig 7K-O.

1. Zhang Z, Hou H, Yu S, Zhou C, Zhang X, Li N, et al. Inflammation-induced mammalian target of rapamycin signaling is essential for retina regeneration. Glia. 2020;68: 111–127.

2. Diekmann H, Kalbhen P, Fischer D. Active mechanistic target of rapamycin plays an ancillary rather than essential role in zebrafish CNS axon regeneration. Front Cell Neurosci. 2015;9. doi:10.3389/fncel.2015.00251

3. He J, Chen J, Wei X, Leng H, Mu H, Cai P, et al. Mammalian Target of Rapamycin Complex 1 Signaling Is Required for the Dedifferentiation From Biliary Cell to Bipotential Progenitor Cell in Zebrafish Liver Regeneration. Hepatology. 2019;70: 2092–2106.

4. Pita-Thomas W, Mahar M, Joshi A, Gan D, Cavalli V. HDAC5 promotes optic nerve regeneration by activating the mTOR pathway. Exp Neurol. 2019;317: 271–283.

5. Volovelsky O, Cohen G, Kenig A, Wasserman G, Dreazen A, Meyuhas O, et al. Phosphorylation of Ribosomal Protein S6 Mediates Mammalian Target of Rapamycin Complex 1-Induced Parathyroid Cell Proliferation in Secondary Hyperparathyroidism. J Am Soc Nephrol. 2016;27: 1091–1101.

6. Zelinka CP, Volkov L, Goodman ZA, Todd L, Palazzo I, Bishop WA, et al. mTor signaling is required for the formation of proliferating Müller glia-derived progenitor cells in the chick retina. Development. 2016;143: 1859–1873.

7. Arenas DJ, Floess K, Kobrin D, Pai R-AL, Srkalovic MB, Tamakloe M-A, et al. Increased mTOR activation in idiopathic multicentric Castleman disease. Blood. 2020;135: 1673–1684.

8. Liu J, Reeves C, Michalak Z, Coppola A, Diehl B, Sisodiya SM, et al. Evidence for mTOR pathway activation in a spectrum of epilepsy-associated pathologies. Acta Neuropathol Commun. 2014;2: 71.

9. Teotia P, Van Hook MJ, Fischer D, Ahmad I. Human retinal ganglion cell axon regeneration by recapitulating developmental mechanisms: effects of recruitment of the mTOR pathway. Development. 2019;146. doi:10.1242/dev.178012

10. Roux PP, Shahbazian D, Vu H, Holz MK, Cohen MS, Taunton J, et al. RAS/ERK signaling promotes site-specific ribosomal protein S6 phosphorylation via RSK and stimulates cap-dependent translation. J Biol Chem. 2007;282: 14056–14064.

11. Biever A, Puighermanal E, Nishi A, David A, Panciatici C, Longueville S, et al. PKA-dependent phosphorylation of ribosomal protein S6 does not correlate with translation efficiency in striatonigral and striatopallidal medium-sized spiny neurons. J Neurosci. 2015;35: 4113–4130.

12. Yano T, Ferlito M, Aponte A, Kuno A, Miura T, Murphy E, et al. Pivotal role of mTORC2 and involvement of ribosomal protein S6 in cardioprotective signaling. Circ Res. 2014;114: 1268–1280.

13. House C, Wettenhall RE, Kemp BE. The influence of basic residues on the substrate specificity of protein kinase C. J Biol Chem. 1987;262: 772–777.

14. Chung J, Kuo CJ, Crabtree GR, Blenis J. Rapamycin-FKBP specifically blocks growth-dependent activation of and signaling by the 70 kd S6 protein kinases. Cell. 1992;69: 1227–1236.

15. Leach LL, Hanovice NJ, George SM, Gabriel AE, Gross JM. The immune response is a critical regulator of zebrafish retinal pigment epithelium regeneration. Proc Natl Acad Sci U S A. 2021;118. doi:10.1073/pnas.2017198118

16. Silva NJ, Nagashima M, Li J, Kakuk-Atkins L, Ashrafzadeh M, Hyde DR, et al. Inflammation and matrix metalloproteinase 9 (Mmp-9) regulate photoreceptor regeneration in adult zebrafish. Glia. 2020;68: 1445–1465.

17. Caldwell LJ, Davies NO, Cavone L, Mysiak KS, Semenova SA, Panula P, et al. Regeneration of Dopaminergic Neurons in Adult Zebrafish Depends on Immune System Activation and Differs for Distinct Populations. J Neurosci. 2019;39: 4694–4713.

18. Kyritsis N, Kizil C, Zocher S, Kroehne V, Kaslin J, Freudenreich D, et al. Acute inflammation initiates the regenerative response in the adult zebrafish brain. Science. 2012;338: 1353–1356.

19. White DT, Sengupta S, Saxena MT, Xu Q, Hanes J, Ding D, et al. Immunomodulation-accelerated neuronal regeneration following selective rod photoreceptor cell ablation in the zebrafish retina. Proceedings of the National Academy of Sciences. 2017;114: E3719.

20. Iribarne M, Hyde DR. Inflammation modulates regeneration in the acute or chronically damaged zebrafish retina.

bioRxiv. 2021. p. 2021.10.06.463331. doi:10.1101/2021.10.06.463331
